# Heterogeneous *Porphyromonas gingivalis* LPS modulates immuno-inflammatory response, antioxidant defense and cytoskeletal dynamics in human gingival fibroblasts

**DOI:** 10.1038/srep29829

**Published:** 2016-08-19

**Authors:** Thanuja D. K. Herath, Richard P. Darveau, Chaminda J. Seneviratne, Cun-Yu Wang, Yu Wang, Lijian Jin

**Affiliations:** 1 National Dental Centre Singapore, Singapore; 2 Faculty of Dentistry, The University of Hong Kong, Hong Kong SAR, China; 3 School of Dentistry, University of Washington, Seattle, USA; 4 Oral Sciences, Faculty of Dentistry, National University of Singapore, Singapore; 5 School of Dentistry, University of California Los Angeles, Los Angeles, USA; 6 Department of Pharmacology & Pharmacy, Li Ka Shing Faculty of Medicine, The University of Hong Kong, Hong Kong SAR, China

## Abstract

Periodontal (gum) disease is a highly prevalent infection and inflammation accounting for the majority of tooth loss in adult population worldwide. *Porphyromonas gingivalis* is a keystone periodontal pathogen and its lipopolysaccharide (PgLPS) acts as a major virulence attribute to the disease. Herein, we deciphered the overall host response of human gingival fibroblasts (HGFs) to two featured isoforms of tetra-acylated PgLPS_1435/1449_ and penta-acylated PgLPS_1690_ with reference to *E. coli* LPS through quantitative proteomics. This study unraveled differentially expressed novel biomarkers of immuno-inflammatory response, antioxidant defense and cytoskeletal dynamics in HGFs. PgLPS_1690_ greatly upregulated inflammatory proteins (e.g. cyclophilin, inducible nitric oxide synthase, annexins, galectin, cathepsins and heat shock proteins), whereas the anti-inflammatory proteins (e.g. Annexin A2 and Annexin A6) were significantly upregulated by PgLPS_1435/1449_. Interestingly, the antioxidants proteins such as mitochondrial manganese-containing superoxide dismutase and peroxiredoxin 5 were only upregulated by PgLPS_1690._ The cytoskeletal rearrangement-related proteins like myosin were differentially regulated by these PgLPS isoforms. The present study gives new insight into the biological properties of *P. gingivalis* LPS lipid A moiety that could critically modulate immuno-inflammatory response, antioxidant defense and cytoskeletal dynamics in HGFs, and thereby enhances our understanding of periodontal pathogenesis.

Periodontitis is characterized by pathogenic plaque biofilm-induced inflammatory destruction of tooth-supporting tissues and alveolar bone, and it remains one of the major global oral health burdens affecting millions of people^1^. *Porphyromonas gingivalis* is a keystone periodontal pathogen^2^,^3^ and its lipopolysaccharides (PgLPS) is a crucial virulence factor in periodontal pathogenesis^4^. The critical structure of LPS is the phosphorylated glucosamine disaccharide with fatty acids lipid A, being a highly variable O-polysaccharide and a well conserved core oligosaccharide^5^. Lipid A is indeed the active core of LPS owing to its specific and highly sensitive recognition by the innate immune system. It is worthy to note that the LPS structure varies widely among Gram-negative bacteria. Alterations in the lipid A domain of PgLPS, such as changes in the number, length and composition of fatty acids attached, and the level of phosphorylation, or subtraction of phosphate or monosaccharide groups, can markedly affect the biological activities of host cells^6^,^7^,^8^.

*P. gingivalis* LPS lipid A structure is one of the most extensively studied molecular structures among oral bacteria due to its remarkable heterogeneity^2^,^3^,^4^,^6^,^7^,^8^,^9^,^10^,^11^. Hence, *P. gingivalis* contains multiple lipid A moieties which structurally and functionally differ from the canonical lipid A structure of *E. coli*. Interestingly, hemin concentration and temperature may influence the expression of lipid A structure of *P. gingivalis* LPS^9^,^11^. Hence, PgLPS alters significantly its lipid A structure under different microenvironmental niches, and presents two predominant isoforms including penta- (LPS_1690_) and tetra-acylated (LPS_1435/1449_)^6^. It has been demonstrated that these isoforms can critically modulate immuno-inflammatory response and the underlying signal transduction cascades^2^,^3^,^10^, and indeed the LPS_1435/1449_ possesses different biological activity from the hexa-acylated *E. coli* LPS^3^,^5^. Notably PgLPS_1690_ activates TLR4-mediated NF-κB signaling pathway, and significantly modulates the expression of IL-6 and IL-8 in human gingival fibroblasts (HGFs)^3^,^10^.

High-throughput proteomics approach provides a new platform to simultaneously analyze the expression pattern of proteins and identify novel biomarkers, and it has been successfully applied to study the pathogenic aspects of *P. gingivalis*^12^,^13^. However, these studies have largely focused on the pathogens *per se*, while less investigated the bacteria-host interactions. Currently, there are only limited proteomics studies on the host response to *P. gingivalis* and its LPS in human monocytes^14^. Considering the foregoing knowledge gap, the present study investigated the host response of HGFs (the predominant cell type in gingival tissues) to the two featured isoforms of *P. gingivalis* LPS_1690_ and LPS_1435/1449_ with reference to the hexa-acylated *E. coli* LPS. In this comprehensive study, we determined both cell-bound and secretory proteomic expression profiles of HGFs using gel- and mass spectrometry-based quantitative proteomic approaches, and the identified novel biomarkers were further validated by various molecular techniques. Differential expression in the key biomarkers of immuno-inflammatory response and cytoskeletal rearrangement in HGFs in response to PgLPS isoforms may critically account for periodontal pathogenesis.

## Results

### Heterogeneous PgLPS modulated the cell-bound proteomic profiles in HGFs

**Figure 1:**
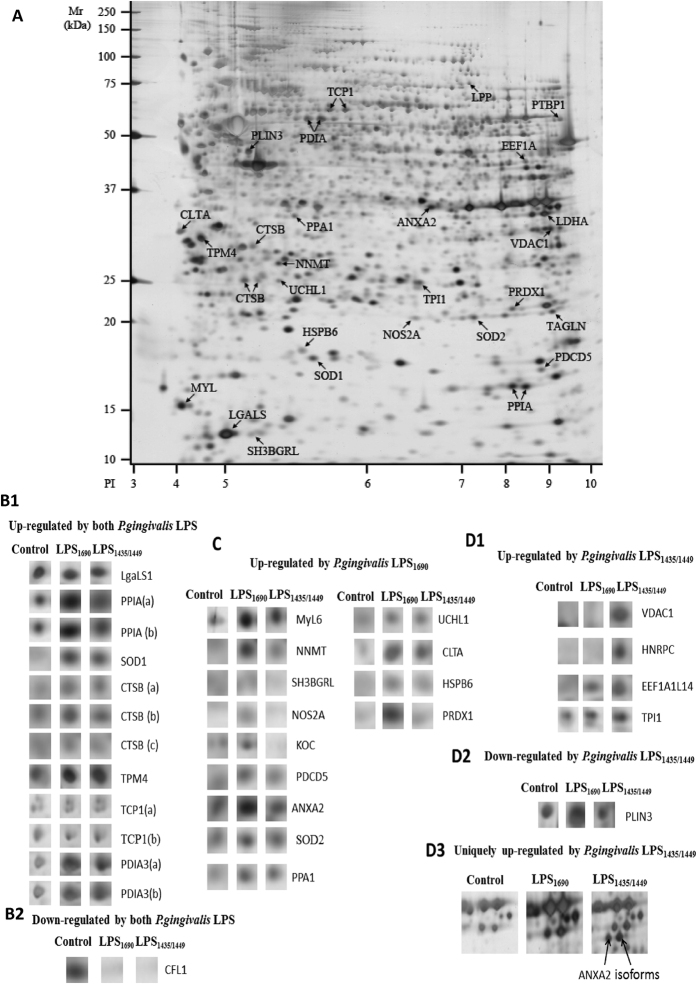
(**A**) Representative 2-DE gel proteomic map of the cellular component of HGFs in response to heterogenous *P. gingivalis* LPS for 24 h. Cellular proteins were seperated via 2-DE, using 24 cm pH 3-10NL IPG strips and homogeneous 12% SDS-PAGE gels. The map was analysed using Image Master 2D platinum software (version.7.0) (Amersham Biosciences). The molecular weight (MW) scale was constructed from protein standards (Bio-Rad Rainbow markers) run alongside the focused strip and the p*I* scale was constructed from the dimensions of the nonlinear pH gradient strips. The map shows the locations of all differntially expressed proteins that were significantly altered by *P. gingivalis* LPS treatment. Labeled protein spots with arrows were equivalent to the gene names specified. Each spot name relates to the data shown in Table S1. (**B**) Significantly up and/or down-regulated proteins in HGFs cellular proteome in response to both *P. gingivalis* LPS_1690_ and LPS_1435/1449_. The density of the protein spots in LPS-treated gels was normalized to that of the corresponding control group. Proteins that upregulated (≥2 folds) (B1) and downregulated (≤0.5 folds) (B2) in *P. gingivalis* LPS-treated groups as compared to the control are shown. Note that proteins labeled with lower case letters (a–c) represent isoforms from the same protein listed in Table S1. (**C**) Significantly upregulated proteins in *P. gingivalis* LPS_1690_ treated cellular proteome. The density of the protein spots in the *P. gingivalis* LPS-treated gels was normalized to the density of the corresponding control group. (**D**) Significantly up- or down-regulated proteins in *P. gingivalis* LPS_1435/1449_ treated cellular proteome. Significantly upregulated proteins (≥2 folds) (D1) and downregulated proteins (≤0.5 folds) (D2) in *P. gingivalis* LPS_1435/1449_ treated groups are shown. The density of the spots in the LPS treated gels was normalized to that of the corresponding control group. Annexin A2 (ANXA2), which was uniquely expressed in *P. gingivalis* LPS_1435/1449_ treated cells, is shown as two isoforms (D3).

PgLPS_1435/1449_ and PgLPS_1690_ -induced proteomic expression in HGFs was analyzed by both gel-based and gel-free proteomics approaches. The study groups consisted of cellular samples without LPS stimulation as the controls, and those treated with PgLPS_1435/1449,_ PgLPS_1690_ and hexa-acylated *E. coli* LPS. Each group showed a typical proteomic signature (Fig. 1). As shown in Table S1, comparative 2-D gel analysis identified a total of 34 differentially expressed protein spots (≥2 folds) between the PgLPS-treated cells and the controls (*P* < 0.05). These differentially expressed proteins encompassed those related to tissue homeostasis including immuno-inflammatory response (e.g. cyclophilin, inducible nitric oxide synthase, annexins, galectin, cathepsins and heat shock proteins) and antioxidant response (e.g. peroxiredoxin, super-oxide dismutase and heat shock proteins). The proteins related to cytoskeletal rearrangement (e.g. myosin) were also differentially expressed in HGFs. Overall, there were 13 upregulated proteins in PgLPS_1690_-treated samples, whereas only six proteins were upregulated by PgLPS_1435/1449_ with reference to the control (Figs 1A, 1B12, 1C and 1D13). Interestingly, proteins included in the antioxidant defense category such as Mn-superoxide dismutase (MnSOD/SOD2) (3.1 folds), human SH3 binding glutamic rich protein (SH3BGRL) (2.2 folds), nitric oxide synthate 2A (NOS2A) (2.6 folds) and peroxiredoxin1 (PRDX1) (4.2 folds) were upregulated in PgLPS_1690_-treated cells as compared to the control. Peptidylprolyl isomerase A/Cyclophilin A (PPIA) existed as two isoforms in PgLPS_1690_-induced proteome with pH range from 7.44–7.68 (designated as the Spots17 and 18 in Table S1). AnnexinA2 (ANXA2) existed as multiple isoforms in PgLPS-induced proteome. It was observed as two unique spots in PgLPS_1435/1449_-stimulated proteome, while another form was upregulated by PgLPS_1690_ (Figs 1A, 1C and 1D3). On the other hand, some proteins were found to be downregulated by PgLPS. For instance, cofilin-1 (CFL1) was downregulated (0.2 folds) by both *P. gingivalis* LPS isoforms. While, both isoforms upregulated eight kinds of proteins such as human galactin-1(LGALS1), cathepsin-B (CTSB), protein disulphide isomerase A (PDIA3) and Cu-Zn SOD1 with reference to the control.

In order to further examine the proteome changes induced by PgLPS stimulation, the cell-bound protein fraction of HGFs was analyzed by gel-free, label-free quantitative LC-MS/MS proteomics. The data of the PgLPS-induced cellular proteins are presented in Table S2. A total of 164 differentially expressed proteins (≥2 folds) in PgLPS-induced cellular proteome were selected for further analysis. Corroborating gel-based proteomics, key protein biomarkers in tissue homeostasis and cytoskeletal rearrangement were differentially expressed (Table S2). For instance, the annexins family such as annexins A1, A2, A5 and A6 were upregulated in PgLPS-induced cellular proteome. There was no significant difference in Annexin A1 and A5 expression between the samples treated by the two PgLPS isoforms. In contrast, annexins A2 and A6 were significantly upregulated in PgLPS_1435/1449_-treated HGFs as compared to the ones treated by PgLPS_1690_.

A number of proteins associated with antioxidant response such as peroxiredoxin, thioredoxin and superoxide dismutase were significantly upregulated in PgLPS-treated cells as compared to the controls. PRDX2 and PRDX4 were similarly upregulated by both isoforms of PgLPS. The expression of PRDX1 was higher in PgLPS_1690_-treated group, whilst PRDX6 was expressed strongly in PgLPS_1435/1449_ group. Interestingly, PRDX5 was only identified in PgLPS_1690_-treated samples. Differential expression of SOD, a group of antioxidants, was found in cells treated by PgLPS. For example, SOD1 was significantly upregulated in both PgLPS-treated groups compared to the controls. Interestingly, MnSOD (SOD2) was upregulated only in PgLPS_1690_-treated samples and no expression was detected in PgLPS_1435/1449_-treated ones. These results corroborated the findings of gel-based proteomics experiments (Fig. 1; Table S1). The levels of heat shock protein (HSPA), the major stress response protein, showed significant increase in cells treated by PgLPS_1690_. Cathepsins which are members of cysteine protease family were shown to be differentially regulated. Among them, the major cysteine peptidase enzyme CTSB was upregulated in PgLPS-treated cells in consistence with the gel-based proteomics results (Table S1, Fig. 1). Interestingly, cathepsin-L1 (CTSL1) was only identified in PgLPS_1690_-treated cells, whereas cathepsin D was upregulated similarly in cells treated by both isoforms of PgLPS. Moreover, the inhibitor of cathepsin B, known as cystatin B (CSTB), was highly upregulated by PgLPS_1690_. The proteins related to cytoskeletal rearrangements were significantly upregulated in PgLPS-treated cellular proteome. For instance, vimentin, filamin and many isoforms of myosin were similarly induced by the two groups of cells treated by isoforms of PgLPS. There was no significant difference in the expression of aforementioned proteins between the two isoforms of PgLPS.

### Heterogeneous PgLPS modulated the profiles of secretory proteins

**Figure 2:**
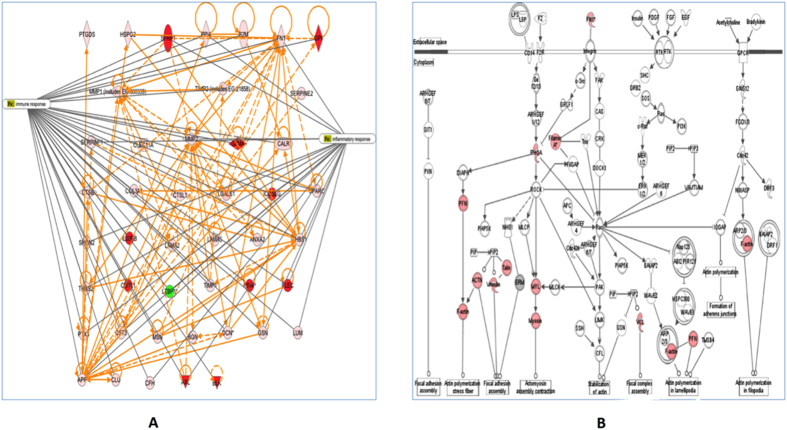
(**A**) Gene ontology and interaction networks of proteins involved in immuno-inflammatory pathway of *P. gingivalis* LPS-induced secretome analysed by Ingenuity Pathways Analysis software (IPA). Red color symbol shows highly upregulated proteins (≥2 folds) compared to untreated cells. Solid lines show direct interaction and dotted lines show indirect interactions. Enzymes: ◊ kinases: ▽; transcription regulators: ⬭ and others: ⬯. (**B**) Gene ontology and interaction networks of proteins involved in extracellular matrix synthesis and signalling of *P. gingivalis* LPS-induced secretome analysed by Ingenuity Pathways Analysis software (IPA). Red color symbol shows highly upregulated proteins (≥2 folds) as compared to the untreated cells. Solid lines show direct interaction and dotted lines show indirect interactions. Enzymes: ◊; kinases: ▽; transcription regulators: ⬭ and others: ⬯.

Proteomic analysis of the secretome of PgLPS-treated HGFs was undertaken using a highly sensitive gel-free LC/MS/MS approach (Table S3). Three independent replicates of three different biological samples were obtained for each group, i.e. PgLPS_1435/1449_- and PgLPS_1690_-treated HGFs and the controls. A total of 90 proteins were differentially induced (≥2 folds) by both isoforms of PgLPS. Moreover, a total of 17 proteins were uniquely induced by PgLPS_1435/1449_, whereas 14 proteins were uniquely induced by PgLPS_1690_. Overall, these identified secretory proteins could be categorized into several subgroups, i.e. immuno-inflammatory mediators, extra cellular matrix proteins and others including (i) cytokines (IL-6 and TGF-β); (ii) proteases such as cathepsin B, cathepsinL1, cystatin C, MMP1–2 and A-Disintegrin-and-Metalloproteinase-domain containing protein-28 (ADAM28); and (iii) extracellular matrix proteins (fibronectin, calreticulin, decorin, laminin and osteonectin) (Table S3, Figs 2A and 2B). Ingenuity pathway analysis was used to generate interaction network of the identified proteins according to immuno-inflammatory response as described previously^15^,^16^ (Fig. 2A). Proteins found to be upregulated in PgLPS-treated HGFs secretome were compared to the control. In addition, proteins were mapped according to their location as extracellular space and cytoplasm proteins which play a major role in the tissue hemostasis (Fig. 2B). These molecular networks are based on the knowledge available on the identified biomarkers.

### Verification of novel biomarkers in tissue homeostasis

**Figure 3:**
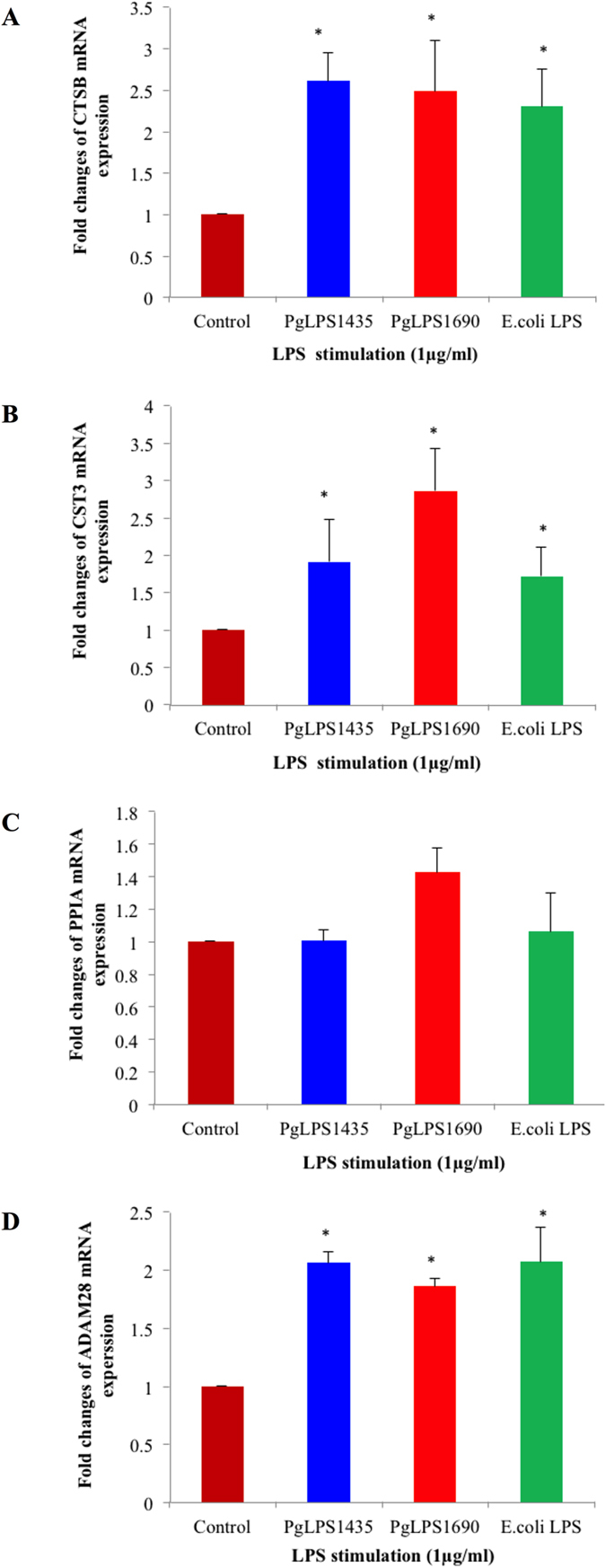
*P. gingivalis* LPS induced gene expression of CTSB, CST3, PPIA and ADAM28 mRNAs in HGFs. Cells were stimulated with *P. gingivalis* LPS and *E. coli* LPS at 1 μg/mL for 24 h. Afterwards, the harvested mRNA was subjected to real time qPCR analysis. Increased gene expression levels of CTSB (**A**), CST3 (**B**), PPIA (**C**) and ADAM28 (**D**) were noted as compared with the internal control. Each bar represents the mean ± SD of three independent experiments with three replicates. *Significant difference from the control with a *P* -value < 0.05.

**Figure 4:**
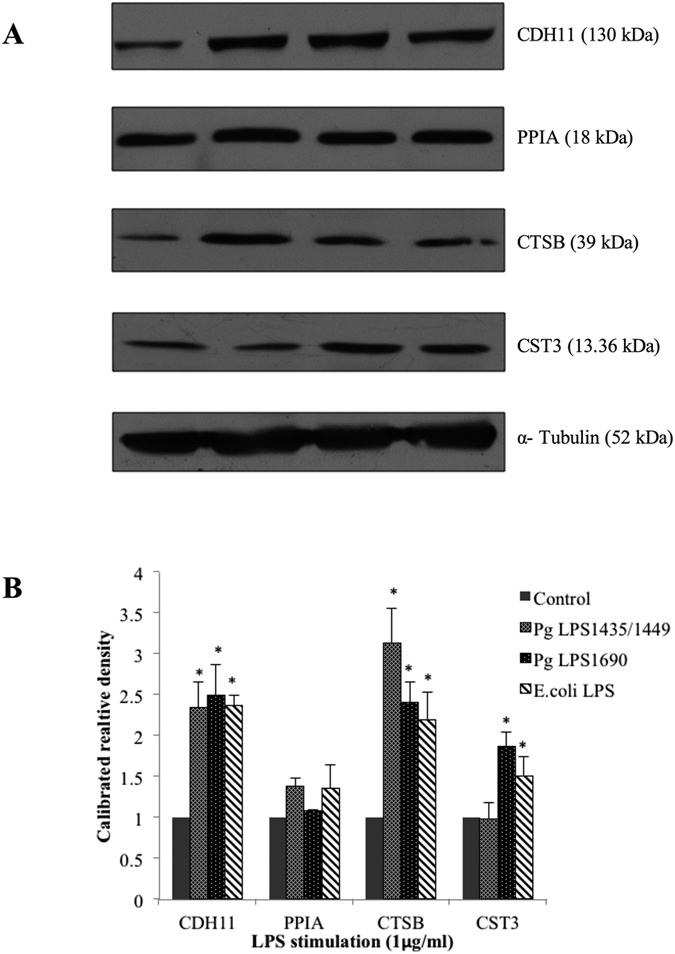
The gene expression of CDH11, PPIA, CTSB and CST3 proteins in *P. gingivalis* LPS- and *E. coli* LPS-stimulated HGFs analysed by Western blot. Confluent cells were treated with *P. gingivalis* LPS and *E. coli* LPS (1 μg/mL) for the indicated time points. 40 μg of homogenized, cellular extracts, were subjected to SDS-PAGE and probed with polyclonal antibodies against CDH11 (1:250), PPIA (1:1000), CTSB (1:1000) and CST3 (1:1000). Blots were reprobed with α-Tubulin to confirm the equal loading in individual samples. (**A**)The fold increase values of proteins as compared with α-Tubulin are shown in the graphs (arbitrary units over control after normalization to the total protein). (**B**) One representative blot is shown from three independent experiments with similar results. ^*^Significant difference with a *P*-value <0.05 as compared with the controls without LPS treatment.

The identified biomarkers related to different functional groups were further examined using real-time qPCR. These included proteins related to tissue homeostasis such as CTSB, cystatin C (CST3), PPIA and ADAM28 Fig. 3). PgLPS significantly induced the expression of CTSB, CST3 and ADAM28. Notably, the expression level of CST3 was higher in PgLPS_1690_-treated cells. On the contrary, there was no significant effect on PPIA mRNA expression in LPS-treated samples. Western blot analysis further validated the expression of protein biomarkers, including cadherin (CDH), CTSB, CST3 and PPIA (Fig. 4). Both isoforms of PgLPS could slightly upregulate the CTSB expression in consistent with the findings of cellular proteomics and secretome ananlysis. PgLPS_1690_ induced the expression of CST3 with reference to the controls.

### Identification of novel biomarkers in antioxidant response

**Figure 5:**
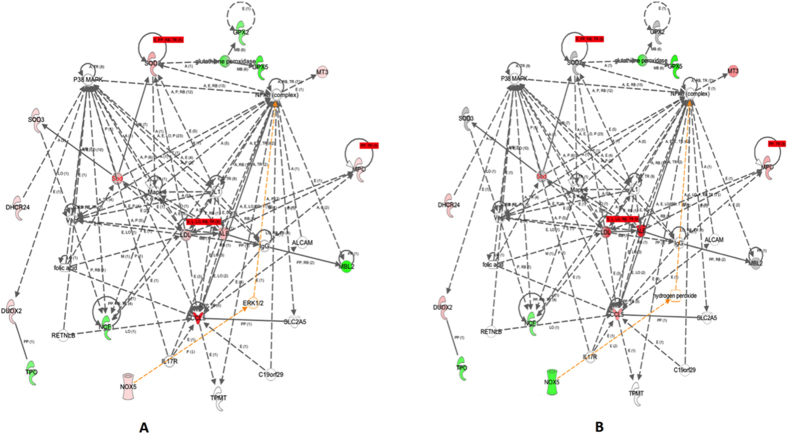
(**A**) Gene ontology and interaction networks of proteins related to oxidative stress and antioxidant defense pathway in *P. gingivalis* LPS_1690_-induced HGFs secretome analysed by Ingenuity Pathways Analysis software. Upregulated genes are shown in red color and down regulated ones are shown in green color. *P. gingivalis* LPS_1690_ markedly induced the antioxidant defense molecules SOD2 (MnSOD), SOD3 and NOX5. (**B**) Gene ontology and interaction networks of proteins related to oxidative stress and antioxidant defense pathway in *P. gingivalis* LPS_1435/1449_-induced HGFs secretome analysed by Ingenuity Pathways Analysis software. Upregulated genes are shown in red color and down regulated ones are shown in green color.

In order to ascertain the effect of heterogeneous PgLPS on oxidative stress response-associated genes, RT^2^ Profiler PCR array consisting of 84 genes related to ROS metabolism, antioxidants and oxygen transporters was performed, and the genes with differential expression (≥1.5 folds) as compared to the controls were further analyzed (Table S4). Interestingly, PgLPS_1690_ markedly upregulated (≥5 folds) the transcript levels of antioxidants, albumin (ALB), peroxidasin like (PXDNL) and MnSOD compared to the controls. Of them, MnSOD was strongly upregulated (fold changes ≥16.0) in PgLPS_1690_-treated cells, whilst no significant upregulation was found in PgLPS_1435/1449_-treated cells (Table S4, Fig. 5). Notably, higher expression of chemokine CCL5 was induced by PgLPS_1690_ (fold changes ≥49.0) as compared to PgLPS_1435/1449_-treated ones.

**Figure 6:**
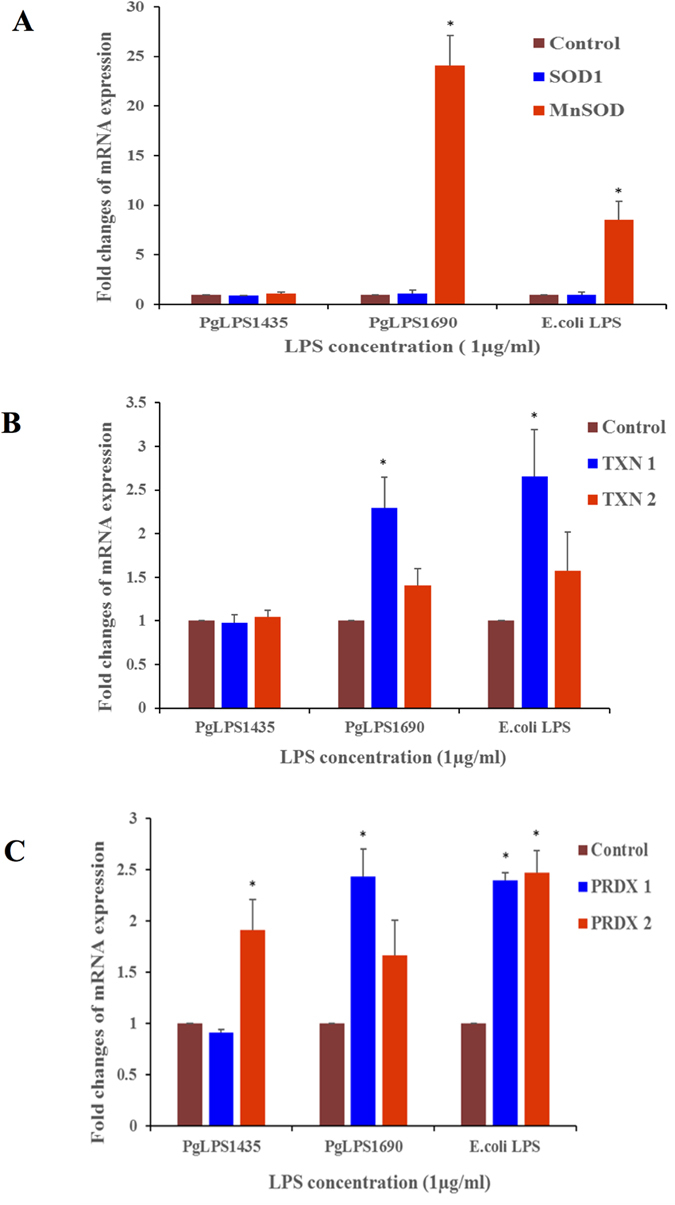
*P. gingivalis* LPS_1690_- and *E. coli* LPS-induced gene expression of MnSOD, TXN1 and PRDX1 mRNAs in HGFs. Cells were stimulated with *P. gingivalis* LPS and *E. coli* LPS at 1 μg/mL for 24 h. After LPS stimulation, harvested RNA was subjected to real-time qPCR analysis. Fold changes of SOD1 and MnSOD (**A**) TXN1 and TXN2 (**B**), PRDX1 and PRDX2 (**C**) gene expression were shown with reference to internal control β-actin. Each bar represents the mean ± SD of three independent experiments with three replicates. *Significant difference with a *P*-value < 0.05 as compared with the controls without LPS treatment.

**Figure 7:**
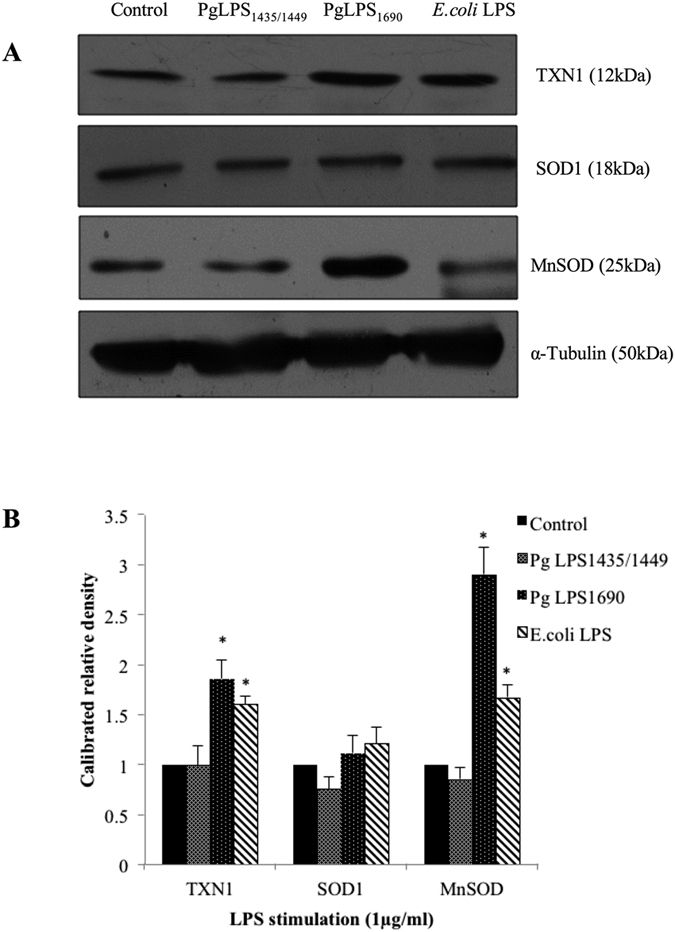
*P. gingivalis* LPS_1690_-induced TXN1 and MnSOD (SOD2) protein expression in HGFs. HGFs were treated with *P. gingivalis* LPS and *E. coli* LPS at 1 μg/ml for 24 h. Protein was pooled from triplicate samples and 40 μg aliquots of total protein extracts were subjected to Western blot analysis. The membranes were probed with rabbit anti-TXN1 mAbs (1:1000), rabbit anti-SOD1 abs (1:1000) and rabbit anti-SOD2/MnSOD mAbs (1:1000) (**A**). For loading control, the membrane was stripped again and incubated with rabbit anti-α-Tubulin mAbs (1:2000). The fold increase values of proteins as compared with α-Tubulin are shown in the graphs (arbitrary units over control after normalization to the total protein) (**B**). One representative blot was shown from three independent experiments with similar results. *Significant difference with a *P*-value <0.05 as compared with the controls without LPS treatment.

In order to validate the results observed in the gene-array, transcript expression of several antioxidant genes in HGFs in response to PgLPS was further evaluated by qPCR. PgLPS_1690_ and *E. coli* LPS (but not PgLPS_1435/1449_) significantly upregulated MnSOD mRNA expression as compared to the control (*P* < 0.05) (Fig. 6). The MnSOD expression reached to the peak at 24 h corroborating the time-dependent observations (Supplementary Fig. S1). On the contrary, no time-dependent increase was found in PgLPS_1435/1449_-treated cells. Furthermore, TXN1 and PRDX1 transcript levels were upregulated in PgLPS_1690_- and *E. coli* LPS-treated cells; whereas PRDX2 transcript expression was significantly upregulated by PgLPS_1435/1449_- and *E. coli* LPS-treated cells (Fig. 6). Besides, TXN2 transcript expression remained unchanged by LPS stimulation. PgLPS_1690_ upregulated the TXN1 transcript expression in a similar manner to MnSOD, although the expression was not as high as the latter (Fig. 6 and Supplementary Fig. S1). Hence, three-fold upregulation of TXN1 was observed at 24 h following PgLPS_1690_ treatment. Moreover, Western blot analysis further confirmed PgLPS_1690_-induced expression of TXN1 and MnSOD at protein level (Fig. 7). These results indicate that PgLPS_1690_ is a potent activator of MnSOD and TXN1 mRNA and protein expression in HGFs.

## Discussion

LPS is a potent stimulator of cellular immune responses. LPS stimulated cells undergo various conformational changes due to differential expression of gene and protein profiles. Therefore, analyzing *P. gingivalis* LPS-induced proteome is of great interest for comprehensive understanding of the host-pathogen interplay. There are no studies in the literature on the differential proteome of *P. gingivalis* LPS induced HGFs, particularly to show the impact of lipid A heterogeneity on the periodontal biology. Therefore, the present study comprehensively investigated the host response of HGFs to heterogeneous PgLPS, using both gel-based and gel-free proteomics approaches. Cell-bound proteome analysis shows that PgLPS could modulate the key biomarkers related to tissue homeostasis including inflammatory and antioxidant responses, and cytoskeleton dynamics (Fig. 1 and Supplementary Tables S1 and S2). Secretory proteome analysis further unravels novel biomarkers related to inflammatory response and extracellular matrix dynamics which may account for the pathogenic behavior of *P. gingivalis* in the pathogenesis of periodontitis (Fig. 2 and Supplementary Table S3). Some key biomarkers discovered in the present study and their potential implications in periodontal pathogenesis are elaborated herein.

Inflammation is one of the key host defense mechanisms and critically contributes to healing response and tissue homeostasis. We have previously demonstrated that the penta-acylated PgLPS_1690_ induces classical pro-inflammatory markers of IL-6 and IL-8 in HGFs^3^,^10^. Taking a step forward, we analyzed the other protein biomarkers associated with immuno-inflammatory response using gel-based and gel-free proteomics approaches. The present study unravels highly upregulated inflammatory biomarkers in cell-bound proteome by PgLPS_1690_ as compared to PgLPS_1435/1449_, such as cyclophilin, iNO synthase, annexins, galectin, cathepsins, and heat shock proteins (Fig. 1 and Supplementary Tables S1 and S2). Cyclophilin A (CyPA) or termed as peptidyl-prolyl isomerase A (PPIA) is an abundant cytosolic protein in the immunophilin family. It is a potent pro-inflammatory molecule and transfers signals between immune and structural cells. It could therefore act as a Damage-associated molecular pattern (DAMP) molecule in inflammation. Interestingly, PPIA could regulate inflammatory response and MMP production through interacting with the extracellular matrix metalloproteinase inducer (EMMPRIN)^17^. In an experimental periodontitis model, it has been demonstrated that both PPIA and EMMPRIN are significantly upregulated in inflamed gingiva with reference to the healthy controls^18^. The present study suggests that PPIA may be an important element in host response to PgLPS that alarms host cells about the invasion of pathogens like *P. gingivalis*. Nitric oxide synthase 2A (iNOS or NOS2A) is another protein that could be upregulated in the proteome of HGFs in response to PgLPS_1690_. NOS2A is important for inflammatory response and acts against invading pathogens^19^. It has been suggested that iNOS may be involved in periodontal pathogenesis^20^. The LPS of common periodontal pathogens could induce the expression of iNOS in various host cells^21^. In addition, *P. gingivalis* LPS induces iNOS production in murine macrophages^22^. Our present study shows that PgLPS_1690_ could induce a stronger inflammatory response as compared to the PgLPS_1435/1449_ in consistence with our previous findings on classical pro-inflammatory cytokines^3^,^10^.

On the contrary, some of the protein markers expressed by HGFs in response to heterogeneous PgLPS are of anti-inflammatory in nature, such as annexin family of proteins (Fig. 1 and Supplementary Tables S1 and S2). Annexins (ANXA) are calcium-dependent phospholipid-binding proteins having diverse roles in cellular funnctions^23^. These proteins are involved in development and resolution of inflammation through pro- and anti-inflammatory effects^24^. Several ANXA proteins such as ANXA1–5 have been extensively investigated in their involvements in immuno-inflammatory pathways^25^. In the present study, PgLPS induced the expression of ANXA1-3 and ANXA6 in HGFs. Of them, ANXA1, ANXA2 and ANXA6 were upregulated by PgLPS_1435/1449_ but not by PgLPS_1690_. In the gel- based cellular proteome experiments, two unique protein spots of ANXA2 with pI value of 8.32–8.41 were identified (Fig. 1D3). It seems that upregulation of these particular annexin proteins in HGFs proteome may reflect a specific effect of PgLPS_1435/1449_. There are other studies in line with this observation, demonstrating the anti-inflammatory activity ofANXA1 protein^26^. ANXA1 specifically targets cytosolic phospholipase A2 via inhibition and suppression of cytokine-induced enzyme activation^26^,^27^. ANXA1 inhibits the expression of other inflammatory mediators such as iNOS or NOS2 in macrophages and inducible cyclooxygenase (COX-2) in activated microglia^28^. Moreover, ANXA1 could inhibit the chemotaxis of neutrophils and monocytes during inflammation^27^,^29^. Therefore, higher expression of ANXA1 in PgLPS_1435/1449_-treated HGFs may account for the inhibition of cytokines by this specific isoform of PgLPS^3^,^10^. Annexin A2 is the heterotetrameric protein with two sub-units of 36-kDa p36 complexed with two 11-kDa S100A10 protein subunits, and it possesses both pro- and anti-inflammatory effects in different circumstances^24^. Further studies are warranted to determine the exact roles of these proteins.

Galectin-1 (LGALS1) is a beta-galactoside-binding protein and plays a crucial role in immuno-inflammatory activities^30^. It is induced by microbial infection and subsequently modulates the development and resolution of innate and adaptive immune responses. Although LGALS1 was upregulated by both isoforms of PgLPS as shown in gel-based cellular proteome cellular proteome, it was more markedly induced by PgLPS_1435/1449_ (23.9 folds) with reference to PgLPS_1690_ (7.1 folds) (Fig. 1, and Supplementary Tables S1 and S2). It has the ability to inhibit the secretion of pro-inflammatory cytokines and reduce prostaglandin E2 secretion and arachidonic acid release from stimulated peritoneal macrophages^31^. In addition, LGALS1 inhibits IL-1β-induced recruitment of neutrophils and their transendothelial migration^32^. It also enables to inhibit *A. actinomycetemcomitans* LPS-induced expression of IL-6 and IL-8^33^. These data suggest that PgLPS_1435/1449_ isoform could paralyze the cytokine network, inhibit leukocyte migration and thereby induce some sort of anti-inflammatory activities^34^. Hence, activation of LGALS1 may be an important determinant for *P. gingivalis* to disguise the activation of host defense, and invade and proliferate in the gingival tissues. These data further confirm the contrasting biological activity of these PgLPS isoforms^3^,^6^,^7^,^10^, suggesting that the shift of penta-acylated lipid A isoform to tetra-acylated lipid A one may be critical for *P. gingivalis* to paralyze the innate host defense in the pathogenesis of periodontal disease^7^.

Secretome refers to a complex set of molecules from living cells. Hence, analysis of these proteins is of great importance owing to their ability to unravel specific cell functions^35^. The present study shows that PgLPS_1435/1449_ and PgLPS_1690_ differentially modulate the expression of MMPs and tissue inhibitors of metalloproteinase (TIMPs). Although both isoforms induced MMP1 and MMP2 as shown in LC/MS analysis, the expression levels were slightly higher in PgLPS_1435/1449_-treated HGFs (Fig. 2A and Supplementary Table S3). In contrast, TIMP levels were differentially regulated by these isoforms, as TIMP1 level was highly upregulated by PgLPS_1435/1449_, whereas TIMP2 was slightly induced in PgLPS_1690_-treated HGFs. The balance between active MMPs and their TIMPs controls the extent of extracellular matrix remodeling, and critically contributes to maintaining oral tissue homeostasis^36^. Disruption of the MMPs-TIMPs balance is a hallmark of many chronic inflammatory diseases like periodontitis, rheumatoid arthritis and vascular diseases^37^. Indeed, the destructive process in periodontitis results from dysregulated tissue homeostasis in connection to MMPs and TIMPs^38^. HGFs are the major stromal cells in gingiva, and play a key role in synthesis and regulation of MMPs and TIMPs. Our recent study indeed shows that PgLPS heterogeneity may account for modulating the dynamics of MMPs and TIMPs in periodontal pathogenesis^39^.

Cathepsins are members of cysteine peptidase family and known to be induced by LPS from Gram-negative bacteria^40^. In the present study, both PgLPS isoforms could induce the expression of cathepsin B (CTSB) and cathepsin D (CSTD) in LC/MS proteome (Supplementary Table S2). Notably, cathepsin L1 (CTSL1) was only present in PgLPS_1690_-treated cellular proteome and highly expressed in the secretome of HGFs as compared to PgLPS_1435/1449_. It is interestingly to note that the inhibitors of cysteine proteases, cystatin B and cystatin C, were significantly upregulated in PgLPS_1690_-treated cellular proteome and secretome (Supplementary Tables S2 and S3). Hence, it seems that PgLPS heterogeneity to a certain extent influences the balance of cysteine proteases and their inhibitors. Furthermore, extracellular matrix (ECM) proteins, such as collagen, fibronectin, laminin, decorin, fibrillin, biglycan and desmin, can be secreted by HGFs in response to PgLPS (Fig. 2B and Supplementary Table S3). HGFs are the main cell type that secretes and regulates the ECM in gingiva^41^. It has also been shown that certain ECM proteins can be induced by *E. coli* LPS in rat chrondrocytes^42^. Although most of the secretory proteins were induced in a similar pattern by the two isoforms of PgLPS, PgLPS_1690_ markedly induced the expression of decorin and biglycan in consistent with a previous study^43^. Hence, the penta-acylated PgLPS_1690_ isoform is certainly a more potent inducer of inflammatory response as compared to its counterpart tetra-acylated PgLPS_1435/1449_ isoform.

We also focused on the protein biomarkers associated with PgLPS induced antioxidant defense system of the HGFs. Cytosolic copper/zinc-containing SOD (Cu, Zn-SOD or SOD1) and mitochondrial manganese-containing SOD (MnSOD or SOD2) are key players in antioxidant host response^44^. In the present study, both Cu/Zn-SOD and MnSOD were identified in the gel-based cell-bound proteome, although the expression of MnSOD was higher in PgLPS_1690_-treated HGFs (Fig. 1 and Supplementary Table S1). Corroborating these findings, the LC/MS analysis further confirmed that MnSOD was only upregulated by PgLPS_1690_ (Supplementary Table S2). Previous studies have also shown that bacterial LPS could induce the anti-oxidant MnSOD in order to protect the cells from oxidative stress through TLR4 and NF-κB signaling pathway^45^. Interestingly, we have recently found that PgLPS_1690_ predominately activates the TLR4-NF-κB signaling in HGFs as compared to the weak activation by PgLPS_1435/1449_^3^. Hence, the former isoform of PgLPS specifically induces MnSOD in HGFs. The peroxiredoxin (PRDX) group of antioxidant proteins were abundantly present in PgLPS-treated cell-bound HGF proteome (Supplementary Tables S1 and S2). PRDX consisting of six isoforms (PRDX1–6) is a novel family of antioxidant proteins involved in oxidative stress protection mechanisms by catalyzing the peroxide reduction of H_2_O_2_, organic hydroperoxides, and peroxynitrites^46^. In the present study, all PRDX isoforms except PRDX3 could be detected in LPS-treated HGFs proteome. PRDX1 was upregulated in the cellular proteome deduced by gel-based approach, and LC/MS analysis confirmed the increased expression of PRDX1 and PRDX4 in PgLPS_1690_-treated cells. PRDX5 could be only identified in PgLPS_1690_-treated cells. On the contrary, PRDX6 was only observed in the secretome of PgLPS_1435/1449_-treated cells (Supplementary Table S3). Although PRDX6 shares some structural and functional properties with other PRDXs, it has some unique features and acts as a bifunctional enzyme with glutathione peroxidase and phospholipase α activity^47^. Therefore, the unique expression of PRDX6 induced by PgLPS_1435/1449_ provides new evidence on the importance of PgLPS heterogeneity in regulating cellular functions. SH3 binding glutamic rich protein (SH3BGRL3) is another antioxidant enzyme highly upregulated by PgLPS_1690_. This well conserved small protein exhibits a great similarity to glutaredoxin1 in the thioredoxin superfamily^48^. Bacterial LPS could upregulate the thioredoxin response as an antioxidant mechanism. These findings show that in contrast to PgLPS_1435/1449_, HGFs enable to identify PgLPS_1690_ and activate sufficient antioxidant response. Taking all the foregoing information into account, it can be suggested that *P. gingivalis* LPS lipid A heterogeneity may play a critical role accounting for the oxidative stress response in HGFs.

Cytoskeleton rearrangement is crucial to maintain the structural integrity of cells in host response to bacterial LPS^49^. LPS-induced re-organization of the actin cytoskeleton has been documented in fibroblasts, endothelial cells, macrophages, and neutrophils^50^. The present study provides further evidence on the cytoskeletal dynamics in the response of HGFs to PgLPS. Notably, several major structural proteins such as myosin, keratin, filamin, vimentin and actin are significantly upregulated by PgLPS-induced proteome as compared to the controls (Supplementary Tables S1 and S2). The actin cytoskeleton is vital for the stability of cell membrane and membrane-dependent cell migration, phagocytosis and division^51^. Striking alteration in the cytoskeleton dynamics occurs in response to LPS due to disorganization of actin, vimentin and tubulin filaments^52^. It is noteworthy that the structural protein cofilin-1(CFL1) is downregulated in PgLPS isoforms-induced gel-based HGF proteome (Fig. 1B). Cofilin, an 18kDa actin-binding protein, is critically involved in the dynamics of cytoskeletal rearrangement of filaments^53^,^54^. It undergoes dephosphorylation in the cytoskeleton rearrangement in response to LPS^51^. The dephosphorylated cofilin is then translocated towards the plasma membrane and binds with F-actin. This enhances the turnover of the actin cytoskeleton and increases the number of actin filaments, thereby leading to rearrangement of actin cytoskeleton. Hence, this study has provided fresh evidence of the critical role of cytoskeleton dynamics in host response to LPS.

It could be hypothesized that the differential behavior of the two featured PgLPS isoforms, PgLPS_1690_ and PgLPS_1435,_ may be largely due to their structural differences which result in differential crosstalk with host cells as demonstrated in previous studies of others and ours^3^,^55^,^56^. On the contrary, some studies argue that the TLR2-active component of PgLPS could result from lipopeptide that cannot be removed by phenol re-extraction procedure, and chemically synthetic lipid A activates only TLR4-mediated signaling pathways^8^. Notably, the recent studies have clarified that PgLPS lipid A *per se* does not activate TLR2 signaling, and the PgLPS activation of TLR2 is most likely due to the presence of covalently attached lipoproteins to the LPS, which helps explain the previous observation on the TLR2 activity induced by the suspected unremovable lipoproteins in PgLPS preparations^57^,^58^. It is now well noted that the lipid A structural heterogeneity of PgLPS significantly reflects *P. gingivalis* phenotypes and their different roles in periodontal health and disease^2^,^6^. Hence, elucidating the underlying mechanisms and major biological pathways involved in *P. gingivalis*-host interactions may lay a foundation for further intensive studies on this keystone pathogen and its exact role in periodontal pathogenesis.

In conclusion, this pioneering study enhances our understanding on the host response of HGFs to the heterogeneous structures of *P. gingivalis* LPS, and uncovers novel protein biomarkers related to immuno-inflammatory response, antioxidant response and cytoskeletal dynamics. Hence, the heterogeneity of *P. gingivalis* LPS lipid A structure could contribute critically to periodontal pathogenesis. It is worthwhile to further validate the current findings in *in vivo* studies using animal model of periodontitis. Moreover, diagnostic and prognostic values of the novel biomarkers derived from this study could be tested in future translational studies.

## Materials and Methods

### Preparation of *P. gingivalis* LPS

*P. gingivalis* heterogeneous LPS isoforms were generated in R.P. Darveau’s Laboratory at the University of Washington as previously described^6^. In brief, both isoforms of *P. gingivalis* LPS_1435/1449_ and *P. gingivalis* LPS_1690_ were prepared from *P. gingivalis* strain 33277. Extraction of LPS was performed by cold MgCl_2_-ethanol procedure as described previously^6^. Further, LPS was treated for removal of endotoxin protein followed by enhanced colloidal gold staining to identify the contaminated proteins. Next, the fatty acid composition was analyzed by Gas chromatographic-mass spectrometry. Then the structural analysis of two forms of *P. gingivalis* LPS_1435/1449_ and *P. gingivalis* LPS_1690_ was carried out by matrix assisted laser desorption ionization time-of-flight mass spectrometry. Ultra purified *E. coli* LPS (JM 83-wild type strain) as the reference control was also provided by R.P. Darveau.

### Cell culture of primary HGFs

Primary HGFs were purchased from Sciencell Research Laboratories (Carlsbad, CA, USA) and cultured according to a previously published protocol^3^,^10^. Cells were maintained in 5 × 10^5^ cells/ml in poly-lysine coated polystyrene flasks (75cm^2^) enriched with fibroblast media containing the basal medium, 2% fetal bovine serum with penicillin/streptomycin (0.01% w/v) and fibroblast growth supplement (FGS)^59^,^60^. Confluent cells were fasted overnight with serum free FM medium-acf-FM, and on the following day, cells were treated with PgLPS_1435/1449_ and PgLPS_1690_ using previously optimized dose of 1 μg/ml for 24 h^10^. *E. coli* LPS (JM 83 strain) was used as the reference for downstream western blot and qPCR analyses as we have described previously^3^.

### Cell-bound proteomics profiles of HGFs using gel-based proteomics

The protein component in the cellular fraction and culture supernatants of HGFs treated with PgLPS at 24 h were used to assess both the cellular proteome and secretome, respectively. Cellular proteins were extracted using mammalian protein extraction buffer together with a cocktail of protease inhibitor (Thermo Scientific, Pierce Biotechnology, Rockford, USA). The mixture was then centrifuged at 3000 rpm for 10 min to remove the cell debris, and the remaining supernatant was used as the total protein homogenate. Next, extracted proteins were subjected to clean-up procedure using 2-D clean up kit (GE Healthcare, Uppsala, Sweden) and re-dissolved in lysis buffer containing 6 M urea, 2 M thiourea, 10% glycerol, 50 mM Tris-HCl at pH 7.8–8.2, 2% *n*-octylglucoside 5 mM TCEP (Tris-2-carboxyethyl phosphine hydrochloride), and 1mM protease inhibitor. Concentration of the cleaned up proteins was estimated using 2-D Quant kit (GE Healthcare, Amersham Biosciences) according to the standard curve method using 2 mg/ml BSA solution as the standard.

### Two-dimensional gel electrophoresis (2-DE)

The protein expression profiles of the purified samples in each experimental group were analyzed by 2-DE approach as previously described^61^,^62^. Equal amounts of proteins (150 μg) from LPS-treated samples and controls were subjected to passive rehydration overnight in 400 μl IEF rehydration buffer containing 7 M urea, 2 M thiourea, 2% CHAPS, 10 mM DTT, and 2% IPG buffer (pH 3–10, non-linear, Bio-Rad) and a trace amount of bromophenol blue. IEF was conducted using Ettan-IPGphor IEF system (Bio-Rad, USA) at 20 °C in a stepwise voltage increment fashion according to a slightly modified protocol^61^,^62^. Following the IEF, strips were equilibrated in 5 ml of equilibration buffer containing 50 mM Tris-HCl at pH 8.8, 6 M urea, 30% glycerol, 2% SDS and 20 mg/mL of DTT (Bio-Rad, USA) with gentle agitation for 15 min to ensure that all sulfhydryl groups were reduced. Next, similar equilibration buffer containing 25 mg/ml of iodoacetamide was used for another 15 min to covalently block protein sulfhydryl groups. Following equilibration, the IPG strips were gently washed with SDS running buffer including 25 mM Tris, 192 mM glycine, 0.1% SDS and subjected to SDS-PAGE. Protean II XL electrophoresis apparatus (Bio-Rad, USA) was used for the second dimension. Briefly, 12% 1.5 mm SDS-PAGE gels were prepared according to the in-house protocol. Next, the equilibrated IEF strips were transferred on to the top of the SDS gel cassettes, and the slots were overlaid with few milliliters of 0.5% low-melting agarose containing trace amount of bromophenol blue. At the same time, 2 mm squares of precision plus protein^TM^ blue standards (Bio-Rad, USA) were inserted at each ends of the focused strip to provide the molecular weight markers. Then the gels were run in the second dimension containing running buffer consists of 25 mM Tris, 192 mM glycine, and 0.1% SDS at pH 8.3. The current set at 40 mA/gel in room temperature until the dye front reached the bottom of the gel. Then, the gels were preceded for the staining procedure. The gels were stained by two different methods, i.e. silver staining and fluorescent SYPRO^®^ Ruby followed by modified silver staining. For the silver staining procedure, slightly modified MS-compatible method was used as described elsewhere^61^,^62^. In a separate set of experiments, gels were stained with fluorescent SYPRO^®^ Ruby as follows. Firstly, the gels were fixed in freshly prepared fixing solution (50% methanol, 10% acetic acid) overnight, and on the next day, they were washed with milliQ water three times for 5 min. Gels were then stained in freshly prepared (1:1 stain with water) SYPRO^®^ Ruby protein gel stain (Bio-Rad, USA) and incubated overnight with gentle shaking in the dark. The gels were destained next day using destaining solution (50% methanol, 12% acetic acid) for 30 min followed by washing with milliQ water and visualized by high performance fluorescent scanner, Typhoon^TM^ 9400 (GE Health care, Uppsala, Sweden) for the detection of protein spots. The reproducibility of the gels was evaluated by the preparation of three biological replicates and three experimental replicates to obtain nine gels for each sample. Image analysis was carried out with the ImageMaster 2-D Platinum soft-ware (GE Healthcare, Uppsala, Sweden) as described previously^61^,^63^. The volume and % of volume were automatically computed, including background subtraction in order to correct for differences in gel staining^61^,^63^. All 2-DE gels were of high quality in terms of resolution and consistency in spot patterns. Differentially expressed protein-spots (≥2 folds) were excised for identification by MALDI-TOF MS/MS.

### MALDI-TOF MS/MS analysis

The spots of interest were excised from preparative gels and destained twice with 100 mL of 50% ACN and 50 mM ammonium bicarbonate at pH 8.0 for 15 min. A third washing was performed with 100 mL of ACN for 10 min and gel pieces were dried for 30 min in a vacuum centrifuge. Trypsin digestion was performed as previously described^61^,^62^. Peptides were analyzed by Voyager-DE STR MALDI-TOF MS (Applied Biosystems, USA) and ABI 4800 MALDI-TOF/TOF tandem mass spectrometer (MS/MS) (Applied Biosystems, USA). An in-house MASCOT v2.1 (Matrix Science) search engine (http://www.matrixscience.com/search_form_select.html) was employed to identify the candidate proteins with reference to the NCBI nr-FASTA database. The searching parameters consist of modifications including Cys as S-carbamidomethyl derivate and Met as oxidized methionine, one missed cleavage site, p*I* 3–10 and a range of protein mass (10 to 500 kDa)^61^,^62^. For both PMF and MS/MS protein identifications, the significant probability scores with a p-value <0.05 were accepted, with both protein and total ion scores ≥95% of C.I. Proteins are named following the annotation in NCBI nr database (Table S1).

### Preparation of cell culture supernatants for secretome analysis

The secreted proteins were isolated from PgLPS-treated cell culture supernatants. In brief, culture supernatants were collected in 15 ml falcon tubes and subjected to filter sterilized (0.2 *μ*m, FP POINT 2-S, Schleicher & Schuell, Whatman, London, Great Britain) to remove the cellular debris. The filtered supernatants were ultracentrifuged using Amicon^R^ ultra centrifugal filter devices with 3000 NMWL (nominal molecular weight limit device) (Millipore, Bedford, MA, USA) to facilitate protein precipitation. This procedure was done at 5000 rpm at 4 °C until the concentrated final volume reached to less than 200 μl. The protein concentration was measured by a modified assay (Bio-Rad, Hercules, CA, USA). Next, according to an established experimental protocol, the samples were resuspended with 6 M urea in 100 mM Tris-HCl (pH 8.0) at 1 μg/μl. 200 mM DTT in 100 mM Tris-HCl were then added and incubated for 60 min, and subsequently 200 mM iodoacetamide (IAA) in Tris-HCl were added. After dilution, the mixture contained 0.6 M urea with an adjusted pH of 8.5. Digestion of proteases was undertaken by addition of 200 ng/mL trypsin in 100 mM Tris-HCl at pH 8.0, and the samples were incubated at 37 °C for 16 h. The reaction was stopped with formic acid at a 5% concentration.

### Cell-bound proteomics profiles using Orbitrap mass spectrometer

The protein samples of cellular fractions were purified with acetone precipitation. Then the samples were sequentially solubilized with 8 M urea in 100 mM Tris buffer, reduced by adding dithiothreitol to 5 mM, and carboxyamidomethylated in 25 mM iodoacetamide. The urea concentration was then diluted to 2 M with 100 mM Tris buffer. After that, the samples were digested with trypsin (Promega Corp.) at ratio of 1:100 (w/w) for 16 h at 37 °C. The digests were desalted and concentrated with STAGE tips and resuspended in 10 μl of 0.1% formic acid for sample injection. All experiments were performed on a linear-ion-trap Orbitrap Velos mass spectrometer (LTQ Orbitrap Velos, Thermo-Scientific) with a nano-electrospray ion source connected to a high performance liquid chromatography (HPLC) consisting of micoflow pump and thermostated micro-autosampler (Finnigan, Thermo-Scientific). A 15 cm fused silica emitter (75 μm I.D., 375 μm O.D.; PicoTip^TM^ emitter, New Objective) was used as analytical column. The emitter was packed in-house with methanol slurry of reverse-phased, fully end-capped Reprosil-Pur C18-AQ 5 μm resin (Dr. Maisch GmbH), using a pressure injection cell operated at 60 bar. Mobile phases consist of (A) 0.1% formic acid and 99.9% water (v/v); (B) 0.1% acetic acid and 99.9% acetonitrile (v/v). The 10 μL of prepared peptide mixture was automatically loaded onto an Agilent Zorbax 300SB-C18 trap column (0.3 mm i.d. × 5 mm length, 5 μm particle size) at a set flow rate of 150 μL/min for 10 min in 2% buffer B, followed by an 85 min gradient from 2 to 40% buffer B at a flow rate of ~300 nL/min. MS analysis was performed using unattended data-dependent acquisition mode in which the mass spectrometer automatically switches between a high resolution survey scan with Orbitrap (resolution = 60,000, m/z range 300 to 1800) followed by collision induced dissociation (CID) with linear ion trap of the twenty most abundant peptides eluting at a given time.

### Data analysis, gene ontology and interactome maps

Specific peptides were identified by searching against the IPI database of Human (v3.72) using the search engine SEQUEST (Protein Discoverer 1.1; Thermo-Scientific). Searches were performed with trypsin specificity (up to two missed cleavages), with oxidation of methionine as the dynamic modification and iodoacetamide derivative of cysteine as the static modification. The mass tolerance for monoisotopic peptide identification was set to 7 ppm and ±0.5 Da for fragment ions. The peptide filtering criteria consisted of Cross-correlation (xcorr) values larger than 2.5, resulting in a false discovery rate of ~5% using a decoy search strategy. Minimum of two peptides per protein was needed for protein identification. The search results were imported into Sieve program (version 1.3; Thermo-Scientific) and peptides were label-free quantified using the area under chromatographic peak from extracted ion chromatograms (XICs) of peptides with default parameters for chromatographic alignment and data framing.

The biological functions, in terms of gene ontology and interaction networks, were analyzed by Ingenuity Pathways Analysis software (IPA) (Ingenuity Systems, California, USA) using the gene-ontology obtained by IPI database (http://www.ebi.ac.uk/IPI). IPA analyzes the data by mapping the identified proteins to a manually curated database of protein interactions from literature which is called Ingenuity Pathways Analysis Knowledge Base^15^,^16^. LC/MS data such as protein identifier and expression ratio between control and *P. gingivalis* LPS treated samples were uploaded into the application as previously described^64^,^65^.

Functional categories were grouped and classified according to molecular and cellular function, as well as their roles in physiological systems such as immuno-inflammatory activity and role in antioxidant defense. Based on the available knowledge-based interactions, the identified proteins were connected with hub-proteins in order to obtain interactome maps^14^,^15^. Molecular network represents the potential upstream and downstream interactions, and the biomarkers were grouped according to their biological functions.

### RNA preparation, reverse transcription and transcriptomic analysis using PCR-array

RNA was extracted and isolated from the homogenized HGFs, and cDNA synthesis was undertaken using RNeasy Mini Kit (Qiagen, Hilden, Germany) as previously described^3^. A pathway focused RT^2^ profiler PCR arrays were performed (SA biosciences, Frederick, MD) to screen a panel of genes related to oxidative stress and antioxidant defense (PAHS-065A). In order to ensure the high quality of cDNA, reverse transcription reactions were performed prior to the array, using RT^2^ First Strand Kit (SuperArray, Frederick, MD). Data analysis was carried with the relative gene expression calculated by the ΔΔCt method in the web-based software package for RT^2^ Profiler PCR array systems (SABiosciences, Frederick, MD, USA). Real-time qPCR was carried out to further confirm the selected genes such as SOD1, MnSOD, TXN1, TXN2, PRDX1 and PRDX2. The primers were designed using Primer 3 software (NCBI) and the fold changes in expression of each gene were calculated using the method with the levels of β-actin as an internal control (Table S5).

### Western blot analysis

To validate the proteomic data, the selected proteins were verified by Western blot analysis as described elsewhere^39^. In brief, 40 μg of total cellular proteins from LPS-treated cells were separated on 10% polyacrylamide gel and proteins were then transferred electrophoretically onto PVDF (Polyvinylidene difluoride) membranes (Roche, USA) by using the Mini-PROTEAN Tetra electrophoresis system and the Mini Trans-Blot transfer system (Bio-Rad, USA). Following the transfer, blots were blocked with protein-free T20 (TBS) blocking buffer (Thermo scientific, USA) at room temperature for 1 h and incubated with primary antibody at 4 °C while shaking overnight. Primary antibodies were all obtained against polyclonal rabbit anti-human antibodies; Cathepsin B (1:1000; Cell signaling), Cystatin C (1:1000; BioVendor), Cyclophilin A (1:1000; Cell signaling), and Cadherin (1:500; Invitrogen). α-Tubulin (1:2000, Cell Signaling) was used as the internal loading control. The primary antibodies were detected by use of horseradish peroxidase (HRP) conjugated goat-anti-rabbit IgG (1:10000, Cell Signaling). The immunoreactive proteins were visualized by the use of ECL plus reagents (SuperSignal^®^ West Pico Chemiluminescent kit, Thermo Scientific, USA) and exposed to X-ray films. The bands detected were scanned on a calibrated densitometer (GS-800, Bio-Rad, Hercules, CA, USA) and the integrated density was quantified through Image J software-based analysis (http://rsb.info.nih.gov/ij/).

### Statistical analysis

All experiments were repeated at least three assays for real-time qPCR and Western blot, and two assays for ELISA. All data were presented as mean ± SD. The statistical significance was determined by student *t*-test, one-way ANOVA and Bonferroni and LSD methods as appropriate. A *P*-value with <0.05 was considered statistically significant. All statistical analysis was performed using a software program (SPSS 19.0, SPSS Inc, Chicago, IL, USA).

## Additional Information

**How to cite this article**: Herath, T. D. K. *et al*. Heterogeneous *Porphyromonas gingivalis* LPS modulates immuno-inflammatory response, antioxidant defense and cytoskeletal dynamics in human gingival fibroblasts. *Sci. Rep*. **6**, 29829; doi: 10.1038/srep29829 (2016).

## Supplementary Material

Supplementary Information
