# Author Correction: Heterogeneous *Porphyromonas gingivalis* LPS modulates immuno-inflammatory response, antioxidant defense and cytoskeletal dynamics in human gingival fibroblasts

**DOI:** 10.1038/s41598-021-81798-0

**Published:** 2021-01-22

**Authors:** Thanuja D. K. Herath, Richard P. Darveau, Chaminda J. Seneviratne, Cun-Yu Wang, Yu Wang, Lijian Jin

**Affiliations:** 1 National Dental Centre Singapore, Singapore, Singapore; 2 Faculty of Dentistry, The University of Hong Kong, Hong Kong SAR, China; 3 School of Dentistry, University of Washington, Seattle, USA; 4 Faculty of Dentistry, Oral Sciences, National University of Singapore, Singapore, Singapore; 5 School of Dentistry, University of California Los Angeles, Los Angeles, USA; 6 Department of Pharmacology & Pharmacy, Li Ka Shing Faculty of Medicine, The University of Hong Kong, Hong Kong SAR, China

Correction to: *Scientific Reports*, 10.1038/srep29829, published online 19 August 2016

This Article contains errors.

In Figure 4A, the band for CDH11 is incorrect. As a result, the Figure legend,

“The gene expression of CDH11, PPIA, CTSB and CST3 proteins in *P. gingivalis* LPS- and *E. coli* LPS-stimulated HGFs analysed by Western blot”

should read:

“The expression of CDH11, PPIA, CTSB and CST3 proteins in *P. gingivalis* LPS- and *E. coli* LPS-stimulated HGFs analysed by Western blot”

The correct Figure 4 and its legend appear below as Figure 1.

**Figure 1:**
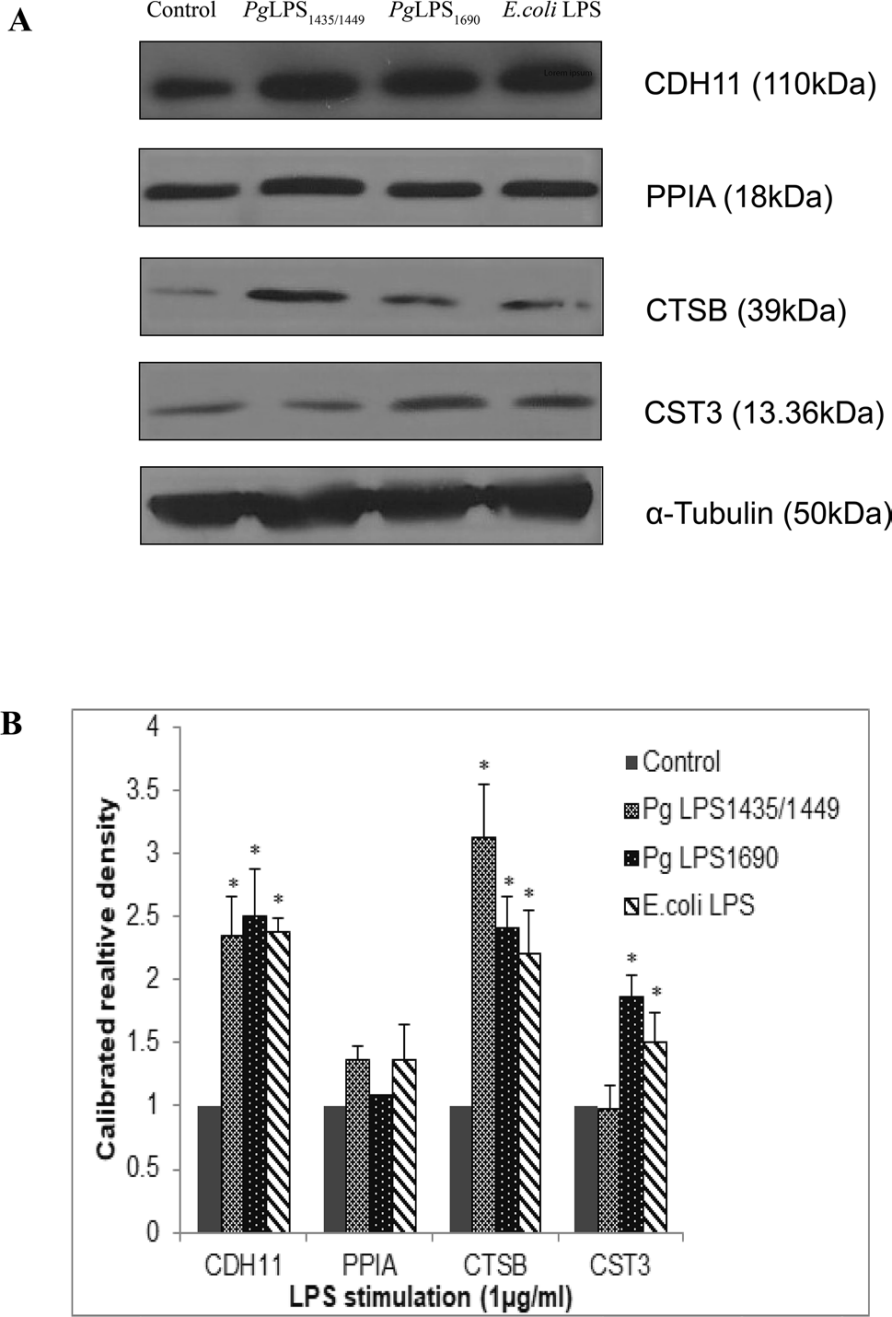
A correct version of the original Figure 4A. The expression of CDH11, PPIA, CTSB and CST3 proteins in *P. gingivalis* LPS- and *E. coli* LPS-stimulated HGFs analysed by Western blot. Confluent cells were treated with *P. gingivalis* LPS and *E. coli* LPS (1 μg/mL) for the indicated time points. 40 μg of homogenized, cellular extracts, were subjected to SDS-PAGE and probed with polyclonal antibodies against CDH11 (1:250), PPIA (1:1000), CTSB (1:1000) and CST3 (1:1000). Blots were reprobed with α-Tubulin to confirm the equal loading in individual samples. (**A**)The fold increase values of proteins as compared with α-Tubulin are shown in the graphs (arbitrary units over control after normalization to the total protein). (**B**) One representative blot is shown from three independent experiments with similar results. ^*^Significant difference with a *P*-value < 0.05 as compared with the controls without LPS treatment.

Furthermore, in Figure 7A, the bands for TXN1 and Tubulin are inadvertently duplicated from Figure 4A’s CST3 band and Tubulin band, respectively, and the band for MnSOD is incorrect.

The correct Figure 7 appears below as Figure 2.

**Figure 2:**
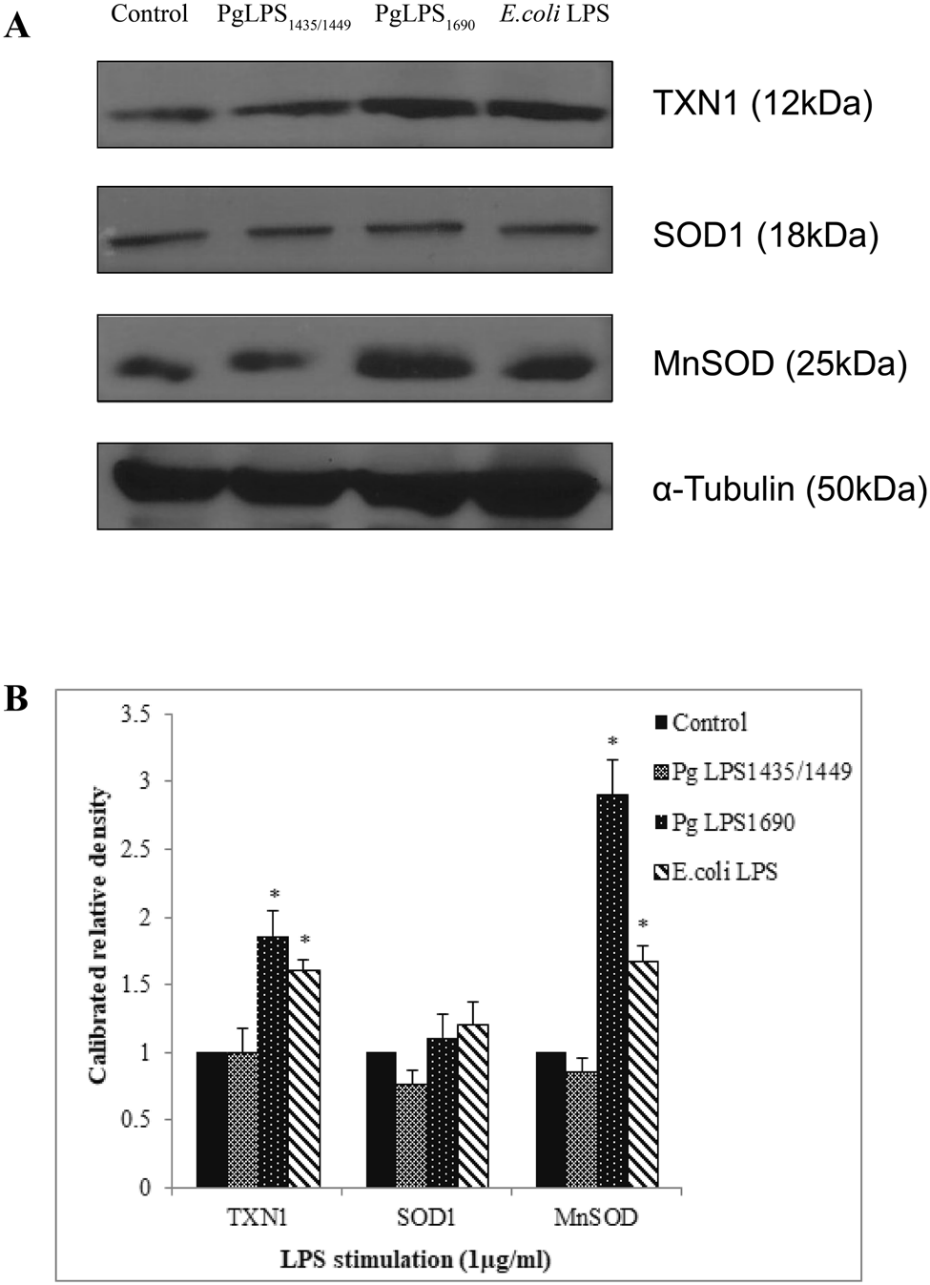
A correct version of the original Figure 7A.

