# Supplementary material for: Heterogeneous Porphyromonas gingivalis LPS modulates immuno-inflammatory response, antioxidant defense and cytoskeletal dynamics in human gingival fibroblasts
Source: Sci Rep. 2016 Aug 19;6:29829. doi: 10.1038/srep29829 (PMC4990928; doi:10.1038/srep29829)
Supplement: Supplementary Information [file srep29829-s1.pdf]

Heterogeneous *Porphyromonas gingivalis* LPS modulates immuno-inflammatory response, antioxidant defense and cytoskeletal dynamics in human gingival fibroblasts

Thanuja D.K. Herath[1,6], Richard P. Darveau[2], Chaminda J. Seneviratne[3], Cun-Yu Wang[4], Yu Wang[5] & Lijian Jin[6]*

[1]National Dental Centre Singapore, Singapore[2], School of Dentistry, University of Washington, Seattle, USA, [3]Oral Sciences, Faculty of Dentistry, National University of Singapore, Singapore, [4]School of Dentistry, University of California Los Angeles, Los Angeles, USA, [5]Department of Pharmacology & Pharmacy, Li Ka Shing Faculty of Medicine, and [6]Faculty of Dentistry, The University of Hong Kong, Hong Kong SAR, China.

*Corresponding author. E-mail: ljjin@hku.hk

A

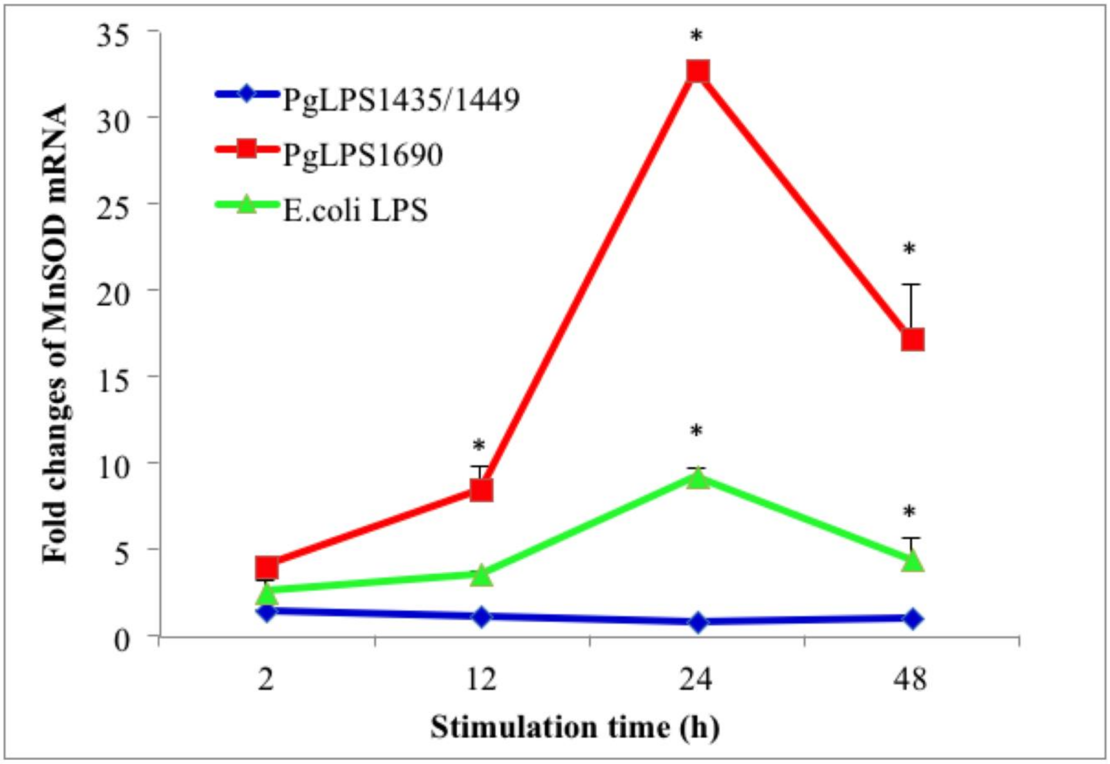

35
30
25
20
15
10
5
0
Fold changes of MnSOD mRNA
PgLPS1435/1449
PgLPS1690
E.coli LPS
*
*
*
*
*
*
2
12
24
48
Stimulation time (h)

B

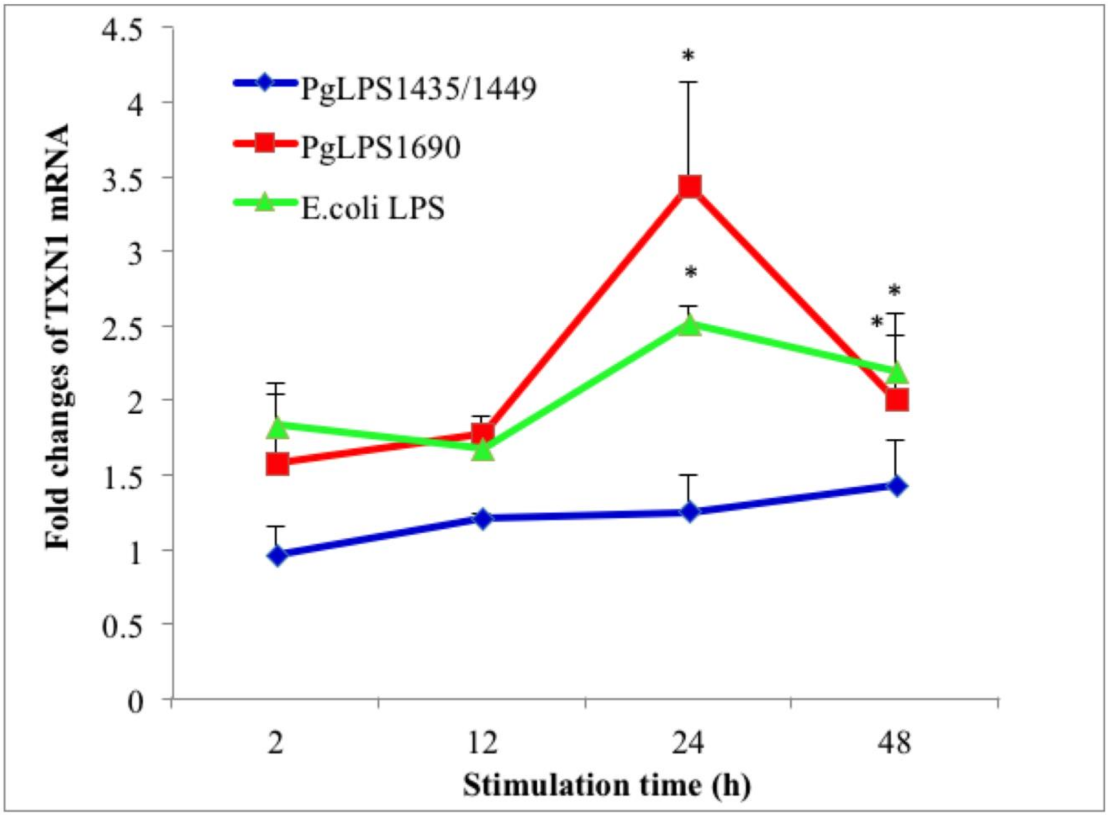

4.5
4
3.5
3
2.5
2
1.5
1
0.5
0
Fold changes of TXN1 mRNA
PgLPS1435/1449
PgLPS1690
E.coli LPS
*
*
*
*
2
12
24
48
Stimulation time (h)

Figure S1. Time-dependent study of the expression of MnSOD and TXN1 genes in *P. gingivalis* LPS treated HGFs. Cells were treated with *P. gingivalis* LPS and *E. coli* LPS at 1 μg/ml for 2 to 48 h in a time-dependent manner. After LPS stimulation, total mRNA was prepared and gene expression of MnSOD (A) and TXN1 (B) mRNAs in the cellular fractions was determined by real-time qPCR. Fold changes in gene expression were shown with reference to internal control β-actin. Each bar represents the mean±SD of three independent experiments with three replicates. *Significant difference with a *P*-value < 0.05 as compared with the controls without LPS treatment.

Spot no	Protein[a]	Accession No	Gene[b]	MW[c] (kDa)	Protein Score	Protein PI[d]	Peptide count[e]	Protein score (CI %)	Fold regulation ↑up↓down	Fold change ± SD[f]		
										Control	$PgLPS_{1435/1449}$	$PgLPS_{1690}$
	**Structural proteins**											
1	Myosin, light polypeptide 6	gi|119617307	MYL6	18310.6	473	4.68	10	100	$PgLPS_{1690}$ ↑	1	2±0.2	6.4±0.4
2	Cofilin-1	gi|5031635	CFL1	18718.7	48	8.22	8	100	both Pg LPS ↓	1	0.2±0.1	0.2±0.2
	**Oxidative stress and antioxidant defense**											
3	Human SH3 Binding Glutamic-rich protein	gi|12230575	SH3BGRL	12766.4	111	5.22	5	100	$PgLPS_{1690}$ ↑	1	1.3±0.1	2.2±0.2
4	Perilipin-3 isoform 3	gi|255958306	PLIN3	45946.3	543	5.28	15	100	$PgLPS_{1435}$ ↓	1	0.5±0.1	3.5±0.2
5	Protein disulfide isomerase	gi|860986	PDIA3	57042.9	418	6.1	23	100	both Pg LPS ↑	1	3.6±0.2	4.4±0.1
6	Protein disulfide isomerase A3 precursor	gi|3659980	PDIA3	57145.9	511	5.98	18	100	both Pg LPS ↑	1	2.4±0.2	3.3±0.1
7	Nitric oxide synthase 2A (inducible)	gi|119571435	NOS2A	111401	52	8.72	15	41.6	$PgLPS_{1690}$ ↑	1	1.2±0.1	2.6±0.2
8	Peroxiredoxin 1	gi|55959887	PRDX1	19134.7	349	6.41	10	100	$PgLPS_{1690}$ ↑	1	1.4±0.2	4.2±0.2
9	Heat shock protein, alpha-	gi|45786106	HSPB6	17198	152	5.95	4	100	$PgLPS_{1690}$ ↑	1	1.4±0.2	2.1±0.3
10	Superoxide dismutase [Cu-Zn]	gi|134611	SOD	16154	161	5.7	5	100	both Pg LPS ↑	1	3.0±0.2	3.3±0.2
	**Protein degradation**											

11	Cathepsin B In Complex With Stefin A	gi|262368170	CTSB	28595.1	209	5.23	8	100	both Pg LPS ↑	1	2.3±0.2	2.4±0.1
12	Lysosomal proteinase cathepsin B	gi|181178	*CTSB*	23632.9	258	5.19	5	100	both Pg LPS ↑	1	2.4±0.1	3.2±0.2
13	Cathepsin B	gi|181178	CTSB	23632.9	185	5.19	6	100	both Pg LPS ↑	1	2.3±0.2	2.3±0.1
	**Enzymes/transcription factors**											
14	Translation elongation factor 1 alpha	gi|15277711	EEF1A1L14	43282.4	287	8.94	6	100	PgLPS$_{1435}$↑	1	4.1±0.1	1.8±0.1
	Mitochondrial											
15	Voltage-dependent anion-selective channel protein 1	gi|4507879	VDAC1	30867.6	249	8.62	13	100	PgLPS$_{1435}$↑	1	3.5±0.1	1.1±0.1
16	Human Manganese Superoxide Dismutase chain A	gi|2780818	SOD2	22290.3	237	6.86	6	100	PgLPS$_{1690}$↑	1	1.2±0.1	3.1±0.3
	**Pro- and anti-apoptotic factors**											
17	Peptidylprolyl isomerase A (cyclophilin A)	gi|3659980	PPIA	18208.9	296	7.44	10	100	both Pg LPS ↑	1	2.3±0.2	5.3±0.2
18	Peptidylprolyl isomerase A (cyclophilin A)	gi|13937981	PPIA	18228	181	7.68	10	100	both Pg LPS ↑	1	2.7±0.3	5.6±0.4
19	Human galectin1	gi|42542977	LGALS1	14868.3	285	5.34	8	100	both Pg LPS ↑	1	2.4±0.1	2.2±0.1
20	Chain A, Annexin A2	gi|56966699	ANXA2	38865.9	365	6.92	17	100	PgLPS$_{1690}$	1	4.5±0.3	2.2±0.3
21	Chain A human annexin A2-	gi|56967118	ANXA2	36630.8	442	8.32	17	100	PgLPS$_{1435}$↑	Unique in PgLPS$_{1435/1449}$		
22	Annexin A2	gi|73909156	ANXA2	40730.9	379	8.41	18	100	PgLPS$_{1435}$↑			
23	Programmed cell death protein 5	gi|6226219	PDCD5	14276.3	30	5.77	5	100	PgLPS$_{1690}$↑	1	1.5±0.3	2±0.2

Miscellaneous

24	TPM4-ALK fusion oncoprotein	gi|10441386	TPM4	27570	347	4.77	11	100	both Pg LPS ↑	1	2.6±0.0	2.7±0.1
25	Human Nicotinamide N-Methyltransferase	gi|118138435	NNMT	31863.1	210	6.5	6	100	$PgLPS_{1690}$↑	1	1.4±0.1	3.5±0.1
26	Clathrin light chain A	gi|6005993	CLTA	27174.1	154	4.43	6	100	$PgLPS_{1690}$↑	1	2.5±0.2	3.6±0.1
27	HumanTriosephosphate Isomerase1	gi|999892	TPI1	26806.8	433	6.51	14	100	$PgLPS_{1435}$↑	1	2.4±0.1	1.5±0.1
28	T-complex protein 1 subunit alpha isoform a	gi|57863257	TCP1	60818.8	329	5.8	16	100	both Pg LPS ↑	1	2.3±0.2	2.3±0.1
29	T-complex protein 1 subunit alpha isoform a	gi|57863257	TCP1	60818.8	493	5.8	20	100	both Pg LPS ↑	1	2.3±0.1	2.1±0.1
30	Polypyrimidine tract-binding protein 1 isoform c	gi|14165466	PTBP1	57356.7	72	9.22	7	98.88	$PgLPS_{1435}$↑	1	2.0±0.2	1.3±0.1
31	Putative RNA binding protein KOC	gi|2105469	KOC	64022.7	212	8.99	16	100	$PgLPS_{1690}$↑	1	1.1±0.2	2.3±0.2
32	Heterogeneous nuclear ribonucleoprotein C (C1/C2)	gi|119586801	HNRPC	19078.8	106	10.22	4	100	$PgLPS_{1435}$↑	1	3.3±0.2	1.2±0.1
33	Pyrophosphatase (inorganic) 1	gi|55962251	PPA1	20254	62	5.07	2	83.54	$PgLPS_{1690}$↑	1	1.6±0.2	2.3±0.2
34	Ubiquitin carboxyl-terminal hydrolase isozyme L1	gi|21361091	UCHL1	25150.6	275	5.33	7	100	$PgLPS_{1690}$↑	1	1.5±0.3	2±0.3

Table S1. Differentially expressed cellular proteins in HGFs in response to *P. gingivalis* $LPS_{1435/1449}$ and LPS_{1690} identified by 2DE-PAGE.

Differentially regulated proteins in HGFs stimulated by two isoforms of *P. gingivalis* LPS, and these proteins correspond to spots whose volume changed (2-fold) after stimulation.

[a]Functional annotation was extracted from NCBInr database.
[b]Genes were named according to the human genome data base (http://www.ncbi.nlm.nih.gov).
[c,d]Apparent mass and p*I* determined from 2-DGE.
[e]Pepetide count indicates the no. of pepetides matched and (%) reports the percent total sequence coverage.
[f]Differential protein expression (fold change) of corresponding protein between LPS-treated and control samples by PD Quest 7.0 (Bio-Rad).

**UniProtKB ID**	**Description**	**Gene**	**Peptides**		**Fold change PgLPS / control**		**P value**
			**PgLPS$_{1435}$**	**PgLPS$_{1690}$**	**PgLPS$_{1435}$**	**PgLPS$_{1690}$**	
**Immuno-inflammatory activity**							
IPI00295741	Cathepsin B	CTSB	2	3	2.9	2.2	2.09E-05
IPI00012887	Cathepsin L1	CTSL1	1	2	no	4.2	0.00127055
IPI00011229	Cathepsin D	CTSD	3	2	2.8	2.5	9.9E-20
IPI00021828	Cystatin_B	CSTB	1	2	3.8	7	0.00050334
IPI00293276	Macrophage migration inhibitory factor	MIF	1	2	2.7	3	0.01730835
IPI00908577	Annexin A1	ANXA1	4	4	2.4	1.8	9.9E-20
IPI00418169	Annexin A2	ANXA2	17	17	2.9	1.8	9.9E-20
IPI00872379	Annexin A5	ANXA5	11	11	2.7	2.7	9.9E-20
IPI00221226	Annexin A6	ANXA6	7	7	2.7	2	9.9E-20
IPI00219219	Galectin_1	LGALS1	2	2	3.4	2.7	4.1193E-05
**Oxidative stress response/antioxidant defense**							
IPI00640741	Peroxiredoxin_1	PRDX1	4	3	3.5	5.6	9.9E-20
IPI00027350	Peroxiredoxin_2	PRDX2	1	2	3.5	3.6	3.5838E-06
IPI00639945	PRDX4 Protein	PRDX4	1	2	2.5	3.6	1.7535E-05
IPI00375306	Peroxiredoxin 5 isoform b	PRDX5		2	No	3.6	4.6742E-08

IPI00220301	Peroxiredoxin_6	PRDX6	4	4	3.0	2.3	5.218E-15
IPI00216298	Thioredoxin	TXN	2	3	2.1	2.2	4.2109E-09
IPI00024919	Thioredoxin_dependent peroxide reductase	PRDX3	2	3	2.8	2.6	1.9772E-08
IPI00106668	perilipin_3 isoform 3	PLIN3	5	4	2.8	2.5	9.9E-20
IPI00218733	Superoxide dismutase [Cu_Zn]	SOD1	2	1	2.6	2.3	1.4206E-06
IPI00022314	Superoxide dismutase [Mn]_ mitochondrial	SOD2	no	2	no	2.8	0.00376528
IPI00219757	Glutathione S_transferase P	GSTP1	2	2	2.7	2.3	4.6111E-08
IPI00909073	Heat shock 70 kDa protein 1	HSPA1	4	4	2.6	3	9.9E-20
IPI00643152	Heat shock 70 kDa protein 1L	HSPA1L	4	4	2.1	2.4	9.9E-20
IPI00002966	HSPA4 Heat shock 70 kDa protein 4	HSPA4	2	2	2.3	2	3.7981E-06
IPI00003362	HSPA5 protein	HSPA5	11	17	2.5	2.2	9.9E-20
IPI00339269	Heat shock 70 kDa protein 6	HSPA6	3	3	2.2	3	9.9E-20
IPI00939595	Heat shock cognate 71 kDa protein	HSPA8	11	13	2.3	2.6	9.9E-20
IPI00922694	Stress_70 protein_ mitochondrial	HSPA9	2	3	2.8	3.4	2.2204E-16
IPI00414676	Heat shock protein HSP 90_beta	HSP90	11	13	2.3	2.4	9.9E-20
IPI00411633	Heat shock protein beta (Fragment)	HSP90AB1	2	3	2.3	2.6	9.9E-20
IPI00917575	heat shock protein_ mitochondrial	HSPD1	7	7	2.6	2.1	9.9E-20
IPI00025512	Heat shock protein beta_1	HSPB1	3	3	2.5	2.5	3.8206E-09
IPI00878551	Protein disulfide_isomerase	PDIA	6	7	2.9	2	9.9E-20
IPI00025252	Protein disulfide_isomerase A3	PDIA3	6	8	2.5	2.2	9.9E-20
IPI00299571	Isoform 2 of Protein disulfide_isomerase A6	PDIA6	4	6	2.2	2.3	9.9E-20
IPI00010796	Protein disulfide_isomerase	P4HB	7	8	2.8	2.2	9.9E-20
IPI00925747	PPIA Peptidyl_prolyl cis_trans isomerase	PPIA	5	5	2.5	2.3	9.9E-20
IPI00646304	Peptidyl_prolyl cis_trans isomerase B	PPIB	4	6	2.2	1.9	9.9E-20
IPI00020599	Calreticulin	CALR	8	7	2.7	1.8	9.9E-20
IPI00290770	T_complex protein 1 subunit gamma isoform b	CCT3	2	4	2.3	2.9	9.9E-20
IPI00797206	CCT8 Protein	CCT8	2	2	1.9	2.4	0.00153141
IPI00020984	Calnexin	CANX	2	3	2.4	2.2	9.9E-20
IPI00032140	SERPINH1 Serpin H1	SERPINH1	6	9	2.6	1.9	9.9E-20
IPI00298268	Prostaglandin G/H synthase 1	PTGS1	3	2	2.6	1.9	9.9E-20

**Cytoskeleton rearrangement**							
IPI00418471	VIM Vimentin	VIM	31	32	2.5	1.9	9.9E-20
IPI00790339	ACTG1 22 kDa protein	ACTG1	2	3	3.0	2.7	9.90E-20
IPI00942659	ACTB 14 kDa protein	ACTB	2	3	3.0	2.7	9.9E-20
IPI00021428	Actin_ alpha skeletal muscle	ACTA1	3	6	2.5	2.5	9.9E-20
IPI00013508	Alpha_actinin_1	ACTN1	9	9	2.4	2.1	9.9E-20
IPI00013808	Alpha_actinin_4	ACTN4	11	11	2.6	2.3	9.9E-20
IPI00174849	Tubulin beta_8 chain B	TUBA	5	5	2.6	2.3	9.9E-20
IPI00646909	Tubulin alpha_8 chain	TUBA8	8	10	2.6	2.5	9.9E-20
IPI00007750	Tubulin alpha_4A chain	TUBA4A	10	13	2.5	2.5	9.9E-20
IPI00180675	Tubulin alpha_1A chain	TUBA1A	13	15	2.5	2.4	9.9E-20
IPI00607818	myosin_14 isoform 1	MYH14	2	1	2.0	2.2	9.9E-20
IPI00033494	Myosin regulatory light chain 12B	MYL12B	2	2	2.0	2.2	9.9E-20
IPI00335168	Myosin light polypeptide 6	MYL6	2	3	2.5	2.4	9.9E-20
IPI00141318	Isoform 1 of Cytoskeleton_associated protein 4	CKAP4	4	3	2.2	1.4	1.2437E-12
IPI00014898	Isoform 1 of Plectin_1	PLEC	35	35	2.3	1.7	9.9E-20
IPI00644576	Filamin A_ alpha	FLNA	16	22	2.2	2.3	9.9E-20
IPI00178352	Filamin_C	FLNC	2	2	3.2	2	0.00715924
IPI00514204	Lamin A	LMNA	4	4	3.8	2.3	9.9E-20
IPI00298994	Talin_1	TLN1	4	2	2.4	2.3	2.7063E-05
IPI00000230	Tropomyosin alpha_1 chain isoform 2	TPM1	2	1	3.1	3	9.9E-20
IPI00903145	Radixin	RDX	2	2	2.2	1.8	9.9E-20
IPI00013991	Tropomyosin beta chain	TPM2	3	2	3.0	2.9	9.9E-20
IPI00219365	Moesin	MSN	2	2	2.2	1.8	9.9E-20
IPI00384282	Cytovillin 2	EZR	2	2	2.2	1.8	9.9E-20
IPI00793184	Isoform 2 of Peripherin	PRPH	3	3	3.3	3	9.9E-20
IPI00291175	Isoform 1 of Vinculin	VCL	3	4	3.1	2.6	1.2836E-06
IPI00216691	Profilin_1	PFN1	5	5	2.8	2.5	9.4594E-12
IPI00550363	Transgelin_2	TAGLN2	4	4	2.4	2.2	9.9E-20

IPI00339225	Isoform 5 of Fibronectin	FN1	4	3	4.1	2	9.9E-20
IPI00339226	Isoform 6 of Fibronectin	FN2	4	3	3.4	1.7	9.9E-20
IPI00339228	Isoform 8 of Fibronectin	FN3	4	4	4.5	1.8	9.9E-20
IPI00942349	Collagen alpha_1(VI) chain	COL6A1	2	2	2.9	1.5	0.00234551
IPI00022200	Collagen alpha_3(VI) chain	COL6A3	10	13	2.6	1.9	9.9E-20
IPI00027463	S100A6 Protein S100_A6	S100A6	2	1	3.5	1.6	1.4612E-09
IPI00257508	Dihydropyrimidinase_related protein 2	PPYSL2	5	5	2.3	2	9.9E-20
**Metabolic enzymes**							
IPI00797270	Triosephosphate isomerase	TPI1P2	8	9	3.1	2.4	9.9E-20
IPI00451401	Triosephosphate isomerase	TPI1P1	7	8	3.1	2.6	9.9E-20
IPI00169383	Phosphoglycerate kinase 1	PGK1	7	4	2.9	2.3	9.9E-20
IPI00549725	Phosphoglycerate mutase 1	PGAM1	3	2	2.8	2.3	1.1102E-16
IPI00759806	Alpha_enolase	ENO1	8	11	2.6	2.3	9.9E-20
IPI00216171	Gamma enolase	ENO2	2	3	2.8	2.3	9.9E-20
IPI00218474	Beta_enolase	ENO3	2	3	2.8	2.4	9.9E-20
IPI00910979	Pyruvate kinase	PKM2	15	15	2.6	2.1	9.9E-20
IPI00795075	Lactate dehydrogenase A chain isoform 5	LDHA	4	4	2.4	2	9.9E-20
IPI00788938	Lactate dehydrogenase	LDHB	2	2	2.4	2.1	9.9E-20
IPI00027497	Glucose_6_phosphate isomerase	GPI	2	2	2.5	2.3	8.6709E-11
IPI00643920	Transketolase	TKT	8	8	2.8	2.1	9.9E-20
IPI00216008	Glucose_6_phosphate 1_dehydrogenase	G6PD	4	6	2.8	2.5	9.9E-20
IPI00031420	UDP-Glucose 6_dehydrogenase	UGDH	2	3	2.4	3.1	0.00136809
IPI00021290	ATP_citrate synthase	ACLY	5	7	2.2	2.3	9.9E-20
IPI00908963	ATP synthase subunit alpha	ATP5A1	5	6	2.8	1.8	9.9E-20
IPI00303476	ATP synthase subunit beta	ATP5B	8	11	2.7	2.1	9.9E-20
IPI00027223.2	Isocitrate dehydrogenase	IDH1	2	2	2.2	2.6	6.864E-05
IPI00952583	Malate dehydrogenase	MDH1	2	2	2.2	1.9	9.9E-20
IPI00291006	Malate dehydrogenase	MDH2	9	10	2.8	2.7	9.9E-20
IPI00018206	Aspartate aminotransferase	GOT2	3	2	2.5	2.4	5.9283E-08

IPI00016801	Glutamate dehydrogenase 1	GLUD1	1	2	2.5	2.8	0.00396268
IPI00004358	Glycogen phosphorylase	PYGB	4	6	2.5	2.9	1.1545E-09
IPI00026781	Fatty acid synthase	FASN	18	19	2.0	2	9.9E-20
**Pro- and anti-apoptotic factors**							
IPI00456750	Niban_like protein 1	FAM129B		2		3.6	1.726E-07
IPI00945782	calmodulin isoform 2	CALM2	2	2	2.8	1.7	9.9E-20
IPI00220740	Isoform 2 of Nucleophosmin	NPM1	2	1	2.0	1.3	5.4068E-14
IPI00289758	Calpain_2 catalytic subunit	CAPN2	2	1	2.1	2.6	9.9E-20
IPI00075248	Calmodulin	CALM2	3	3	2.9	1.8	9.9E-20
IPI00009236	Isoform Alpha of Caveolin_1	CAV1	2	2	3.2	2	9.9E-20
IPI00216088	Cellular retinoic acid_binding protein 2	CRABP2	3	3	3.1	4	0.04600809
IPI00216308	Voltage_dependent anion_selective channel protein 1	VDAC1	3	4	2.2	1	2.5761E-12
IPI00024145	Voltage_dependent anion_selective channel protein 2	VDAC2	2	1	2.3	1.8	9.9E-20
**Signal transduction**							
IPI00946911	Putative uncharacterized protein RAB7A	RAB7A	2	1	2.2	2.4	4.9079E-08
IPI00016342	Ras-related protein Rab_7a	RAB7A	3	2	2.2	2.2	9.992E-16
IPI00024282	Ras-related protein Rab_8B	RAB8B	2	1	-	16.8	9.9E-20
IPI00219446	Phosphatidylethanolamine_binding protein 1	PEBP1	2	1	2.4	2.5	2.5383E-06
IPI00027434	Rho-related GTP_binding protein RhoC	RHOC	2	5	3.0	2.4	9.9763E-11
IPI00478231	Transforming protein RhoA	RHOA	2	5	3.0	2.4	9.9763E-11
IPI00031461	Rab GDP dissociation inhibitor beta	GDI2	2	3	2.7	2.9	6.2343E-08
IPI00552834	Ras homolog gene family_ member C	RhoC	2	2	3.0	2.4	9.9E-20
IPI00848226	Guanine nucleotide_binding protein subunit beta_2	GNB2L1	5	5	2.0	1.9	9.9E-20
**Translation and protein synthesis**							
IPI00396485.3	Elongation factor 1_alpha 1	EEF1A1	7	7	2.6	1.4	9.9E-20
IPI00014424.1	Elongation factor 1_alpha 2	EEFIA2	2	2	2.6	2.6	9.9E-20
IPI00328328.3	Isoform 1 of Eukaryotic initiation factor 4A_II	EIF4A2	3	3	2.6	2.3	9.9E-20
IPI00376005.2	Eukaryotic translation initiation factor 5A_1	EIF5A1	5	3	2.5	1.6	9.9E-20

IPI00006935.3	Eukaryotic translation initiation factor 5A_2	EIF5A2	3	2	2.9	1.7	9.9E-20
IPI00855924.1	Eukaryotic translation initiation factor 5A_1	EIF5AL1	4	2	2.6	1.7	9.9E-20
IPI00186290.6	Elongation factor 2	EEF2	8	9	2.2	2.2	9.9E-20
IPI00789435.2	Elongation factor 1_delta isoform 3	EEF1D	2	3	2.6	2.4	9.9E-20
IPI00003815.3	Rho GDP_dissociation inhibitor 1	ARHGDIA	3	2	2.4	1.8	3.3235E-10
IPI00221224.6	Aminopeptidase N	ANPEP	10	7	2.8	1.8	9.9E-20
IPI00026260.1	Isoform 1 of Nucleoside diphosphate kinase B	NME2	3	3	2.9	2.4	9.9E-20
IPI00604590.3	Nucleoside diphosphate kinase	NME2	3	4	2.9	2.9	9.9E-20
IPI00295386.7	Carbonyl reductase 1	CBR1	3	2	2.5	2.3	2.1986E-08
IPI00176903.2	Polymerase I and transcript release factor	PTRF	2	3	2.1	1.4	9.9E-20
IPI00008530.1	RPLP0 60S acidic ribosomal protein P0	RPLP0	4	2	2.2	2	9.9E-20
IPI00926202.1	RPL15 20 kDa protein	RPL15	2	2	1.8	1.8	0.29751604
IPI00008529.1	60S acidic ribosomal protein P2	RPLP2	4	4	2.2	1.7	7.2095E-06
IPI00021840.1	40S ribosomal protein S6	RPS6	2	2	1.6	1.6	0.03311765
IPI00939811.1	RPS3 Ribosomal protein S3	RPS3	3	4	1.9	2	9.9E-20
IPI00216592.2	Heterogeneous nuclear ribonucleoproteins C1/C2	HNRNPC	2	2	1.5	0.6	4.3592E-06
IPI00216049.1	Heterogeneous nuclear ribonucleoprotein K	HNRNPK	4	4	2.0	1.6	9.9E-20
IPI00023748.3	Nascent polypeptide_associated complex subunit alpha	NACA	2	2	2.4	2.3	9.9E-20
IPI00550069.3	RNH1 Ribonuclease inhibitor	RNH1	7	6	2.5	2.3	1.0059E-13
	Micellaneous functions						
IPI00297160.4	Isoform 12 of CD44 antigen	CD44	2	1	3.0	2.2	1.2212E-15
IPI00021766.5	Isoform 1 of Reticulon_4	RTN4	2	2	3.0	5.1	4.0433E-10
IPI00815745.2	DBI Diazepam binding inhibitor_ splice form 1A	DBI	2	2	2.8	2.3	4.2055E-08
IPI00556376.2	Collapsin response mediator protein 1 isoform 1	CRMP1	2	2	2.6	2	9.9E-20
IPI00411639.1	Laminin receptor_like protein LAMRL5	RPSA	2	2	2.1	1.9	9.9E-20
IPI00010951.2	Epiplakin	EPPK1	2	2	2.4	1.9	9.9E-20
IPI00101037.3	Reticulocalbin_3	RCN3	3	1	3.1	3.2	2.2561E-05
IPI00217906.3	Guanine nucleotide_binding protein G(i) subunit alpha_2	GNAI2	2	2	2.3	2.3	9.9E-20

IPI00010896.3	Chloride intracellular channel protein 1	CLIC1	2	2	2.6	2.6	2.4491E-07
IPI00106642.4	Dihydropyrimidinase_like 2	SDF2L1	4	4	2.3	2	9.9E-20
IPI00555577.1	Thy_1 cell surface antigen variant (Fragment)	THY1	4	3	3.2	2.7	8.5487E-15
IPI00016610.2	Poly(rC)_binding protein 1	PCBP1	3	3	2.7	2.1	3.4565E-07
IPI00015842.1	Reticulocalbin_1	RCN1	3	3	2.4	1.7	6.3079E-07
IPI00022774.3	Transitional endoplasmic reticulum ATPase	VCP	3	4	2.5	2	9.9E-20
IPI00795292.1	Isoform 3 of Nucleoside diphosphate kinase B	NME2	3	4	2.9	2.9	9.9E-20
IPI00908755.1	Ubiquitin_activating enzyme E1	UBA1	2	2	2.5	2	3.394E-13
IPI00024067.4	Isoform 1 of Clathrin heavy chain 1	CLTC	6	8	2.2	2.1	9.9E-20
IPI00021812.2	Neuroblast differentiation_associated protein AHNAK	AHNAK	2	2	2.3	0.2	9.9E-20
IPI00011454	Isoform 2 of Neutral alpha_glucosidase AB	GANAB	2	2	2.6	2.7	2.0668E-06
IPI00914566	Farnesyl pyrophosphate synthase	FDPS	2	2	2.6	1.9	9.2379E-06

Table S2. Differentially expressed cellular proteins in HGFs in response to *P. gingivalis* $LPS_{1435/1449}$ and LPS_{1690} identified by LC-MS/MS approach.

Uniprot ID	Description	Gene	Peptides		Fold change Pg LPS / control		P Value
			$PgLPS_{1435/1449}$	$PgLPS_{1690}$	$PgLPS_{1435/1449}$	$PgLPS_{1690}$	
	**Immuno-inflammatory activity**						
IPI00023673	Galectin-3_binding protein	LGALS3BP	4	6	9.5	7.5	5.63E-07
IPI00219219	Galectin-1	LGALS1	4	3	23.9	7.1	0.093671
IPI00451401	Triosephosphate isomerase	TPI1	2	2	8.1	5.0	7.39E-06
IPI00004656	Beta-2-microglobulin	B2M	3	2	2.2	6.6	0.008975
IPI00025252	Protein disulfide_isomerase A3	PDIA3	3	2	2.8	4.9	0.018986
IPI00003865	Isoform 1 of Heat shock cognate 71 kDa protein	HSPA8	3	2	2.5	4.1	0.094634
IPI00295741	Cathepsin B	CTSB	9	7	10.5	9.6	9.9E-20
IPI00012887	Cathepsin L1	CTSL1	4	4	6.2	14.8	0.006745
IPI00305477	Cystatin_SN	CST1	2	2	3.4	4.6	0.000109
IPI00032293	Cystatin C	CST3	3	3	6.2	10.1	0.212841
IPI00018219	Transforming growth factor_beta	TGFBI	2	2	2.8	3.6	0.032882
IPI00894247	Latent-transforming growth factor beta-binding protein	LTBP1	3	3	2.0	1.3	0.403168
IPI00455315	Isoform 1 of Annexin A2	ANXA2	5	4	5.0	8.3	4.76E-05
IPI00008561	Matrix metalloproteinase 1	MMP1	12	12	8.1	6.3	9.9E-20
IPI00027780	Matrix metalloproteinase 2	MMP2	14	15	6.8	4.0	9.9E-20
IPI00032292	Metalloproteinase inhibitor 1	TIMP1	8	8	13.1	7.1	9.9E-20
IPI00027166	Metalloproteinase inhibitor 2	TIMP2	4	3	3.2	4.3	3.53E-07
IPI00007709	ADAM28 Isoform 1	ADAM28	2	2	5.8	4.7	0.001652
	**ECM matrix proteins**						
IPI00020599	Calreticulin	CALR	4	5	6.2	329.0	0.042337
IPI00219403	Isoform D of Decorin	DCN	4	4	4.5	142.2	7.29E-09
IPI00219400	Isoform B of Decorin	DCN	3	3	4.3	118.6	1.6E-07
IPI00942349	Collagen alpha-1(VI) chain	COL6A1	11	9	6.1	8.0	9.9E-20
IPI00915389	Protein COL6A2	COL6A2	2	2	3.0	4.1	1.57E-05

IPI00871621	COL6A3	COL6A3	37	34	6.4	6.0	9.9E-20
IPI00909936	Collagen alpha-2(I) chain	COL1A2	4	4	5.9	7.9	9.9E-20
IPI00299738	Procollagen C-endopeptidase enhancer 1	PCOLCE	11	6	4.7	5.4	1.97E-12
IPI00942809	LAMA2	LAMA2	3	1	1.5	3.2	0.044728
IPI00852737	LAMB1 95 kDa protein	LAMB1	5	5	7.2	11.3	9.9E-20
IPI00296922	Laminin subunit beta-2	LAMB2	3	4	4.5	9.0	0.000124
IPI00022418	Isoform 1 of Fibronectin	FN1	19	19	6.0	5.5	9.9E-20
IPI00019439	Isoform 1 of Fibrillin-2	FBLN2	6	5	1.4	4.7	0.473587
IPI00296534	Isoform D of Fibulin-1	FBLN1	15	13	5.7	12.9	9.9E-20
IPI00014572	Secreted protein- acidic- cysteine-rich-isoform CRA-a	SPARC	12	12	4.7	7.7	9.9E-20
IPI00006114	plasminogen activator inhibitor-1 isoform 2 precursor	SERPINF1	11	10	3.9	3.7	3.19E-10
IPI00009890	SERPINE2	SERPINE2	10	10	4.7	6.9	9.9E-20
IPI00010790	Biglycan	BGN	5	5	5.4	15.0	9.9E-20
IPI00018769	THBS2 Thrombospondin-2	THBS2	2	2	3.6	3.5	0.044504
IPI00296099	THBS1 Thrombospondin-1	THBS1	8	7	5.0	8.5	1.7E-06
IPI00939199	ABI3BP	ABI3BP	6	6	3.0	4.6	1.06E-10
IPI00020986	Lumican	LUM	7	7	4.2	7.0	3.18E-10
IPI00384542	Isoform 2 of Nidogen-1	NID1	5	2	6.0	7.7	9.9E-20
IPI00028908	Isoform 1 of Nidogen-2	NID2	6	6	6.7	11.0	1.96E-12
IPI00465084	Desmin	DES	4	3	0.9	2.6	0.999998
IPI00006662	Apolipoprotein D	APOD	7	7	5.6	3.7	2.48E-13
IPI00297284	insulin_like growth factor_binding protein 2 precursor	IGFBP2	9	8	2.0	1.4	0.0713022
IPI00305380	Insulin_like growth factor_binding protein 4	IGFBP4	4	4	3.3	4.7	0.045506
IPI00016915	Insulin_like growth factor_binding protein 7	IGFBP7	7	5	6.1	3.4	0.005738
IPI00045396	Isoform 2 of Calumenin	CALU	4	3	5.2	6.7	9.9E-20
IPI00295542	Nucleobindin-1	NUCB1	4	5	5.1	4.5	4.88E-15
IPI00003351	Isoform 1 of Extracellular matrix protein 1	ECM1	4	3	4.4	13.1	0.002005
IPI00024284	heparan sulfate proteoglycan core protein	HSPG2	3	4	4.6	4.7	3.8E-07
IPI00029568	Pentraxin_related protein PTX3	PTX3	3	3	3.8	2.6	0.000268
IPI00010796	Protein disulfide_isomerase	P4HB	3	2	7.2	4.9	4.93E-12

IPI00026314	Isoform 1 of Gelsolin	GSN	3	4	2.7	3.1	3.11E-11
IPI00033466	C type lectin domain family 11 member A	CLEC11A	2	3	7.3	99.6	9.9E-20
IPI00921614	EF-hand calcium-binding domain-containing protein 8	EFCAB6	2	2	3.5	5.8	0.038821
IPI00022974	Prolactin-inducible protein	PIP	2	2	4.4	4.5	0.003077
IPI00216003	Cullin-5	CUL5	2	2	4.2	6.6	0.047657
IPI00418220	Isoform 1 of Zinc finger protein ZFAT	ZFAT	2	2	16.4	13.4	0.009872
IPI00001453	Alpha-internexin	INA	2	2	21.2	27.8	0.013897
IPI00456429	ubiquitin and ribosomal protein L40 precursor	UBA52	2	2	4.1	7.3	9.9E-20
IPI00291262	Isoform 1 of Clusterin	CLU	2	2	1.6	1.6	0.420506
IPI00303335	Nebulin	NEB	2	2	0.8	25.3	0.919507
IPI00298285	Isoform 1 of Receptor tyrosine-protein kinase erbB-3	ERBB3	2	2	2.5	4.1	0.003232
IPI00550746	Nuclear migration protein nudC	NUDC	2	1	2.9	4.3	0.000583
IPI00887675	golgin subfamily A member 6-like protein 1	GOLGA6L1	2	1	5.1	1.9	4.18E-06
IPI00017367	Radixin- isoform CRA_a	RDX	2	1	3.6	3.8	1.83E-09
IPI00219365	Moesin	MSN	2	2	3.6	3.7	1.83E-09
IPI00305975	Spondin-2	SPON2	2	3	13.4	4.4	0.005584
IPI00513767	Prostaglandin D2 synthase 21kDa	PTGDS	2	2	5.8	5.8	9.9E-13
IPI00945507	Putative uncharacterized protein SUCLG2	SUCLG2	2	2	8.3	12.0	2.64E-10
IPI00418780	Isoform 3 of Regulator of G-protein signaling 3	RGS3	2	1	12.1	9.9	0.00844
IPI00006196	Isoform 2 of Nuclear mitotic apparatus protein 1	NUMA1	2	2	8.0	15.5	1.64E-09
IPI00477355	KIF1-binding protein	KIAA	2	1	0.8	2.1	0.798442
IPI00914602	amyloid beta A4 protein	APP	2	1	6.5	1.8	1.01E-06
IPI00646748	Tropomyosin 2	TPM2	2	1	5.8	3.9	9.9E-20
IPI00029739	Isoform 1 of Complement factor H	CFH	2	1	5.9	4.1	0.001987
IPI00220625	Isoform 3 of A_kinase anchor protein 9	AKAP9	2	1	3.8	5.0	1.01E-05
IPI00871702	Isoform 1 of Dysferlin	DYSF	2	1	2.7	2.4	3.05E-11
IPI00792713	Isoform 2	WHSC1L1	2	1	3.8	1.9	7.85E-06
IPI00298961	Exportin-1	XPO1	2	1	7.5	8.9	3.47E-07
IPI00745313	Isoform 1 of Adipocyte enhancer-binding protein 1	AEBP1	2	2	0.9	0.9	0.778485
IPI00022465	Isoform 1 of Citron Rho-interacting kinase	CIT	2	1	2.3	2.9	0.006317

IPI00940990	Dickkopf-related protein 3	DKK3	6	6	10.8	245.3	4.47E-08
	**Miscellaneous**						
IPI00219757	Glutathione S-transferase P	GSTP1	2	2	1.2	2.2	0.005345
IPI00465248	Isoform alpha-enolase of Alpha-enolase	ENO1	6	4	2.4	2.8	0.000497
IPI00218474	Isoform 1 of Beta-enolase	ENO3	2	1	2.2	2.9	9.96E-05
IPI00217966	Isoform 1 of L-lactate dehydrogenase A chain	LDHA	6	3	1.6	2.9	0.946742
IPI00788938	lactate dehydrogenase	LDHB	2	2	1.6	5.8	0.642434
IPI00549725	Phosphoglycerate mutase 1	PGAM1	2	2	7.0	4.7	1.23E-08
IPI00642888	Isoform E of Chloride transport protein 6	CLCN6	2	2	13.0	17.4	0.000475
IPI00465439	Fructose-bisphosphate aldolase A	ALDOA	2	2	7.8	3.5	0.003169
	**Unique proteins induced by *P. gingivalis* LPS$_{1435/1449}$**	**Gene**	**Coverage**	**Peptides**	**Amino acids**	**MW (kDa)**	**Score**
IPI00097434	Interleukin-20 receptor subunit beta	IL20RB	7.4	2	311	35.1	21.84
IPI00398443	Isoform 3 of Serine/threonine-protein kinase WNK4	WNK4	1.97	2	1165	126.5	13.54
IPI00306718	Synaptonemal complex protein 2	SYCP2	1.44	2	1530	175.5	10.23
IPI00012503	Isoform Sap-mu-0 of Proactivator polypeptide	PSAP	2.86	2	524	58.1	10.2
IPI00783524	MLH1-Ex(15-18) isoform	MLH1	10.61	2	179	20.3	9.92
IPI00017704	Coactosin-like protein	COTL1	11.27	2	142	15.9	9.74
IPI00909711	Vinculin	VCL	6.42	2	327	36.2	8.48
IPI00398776	Isoform 7 of Plectin-1	PLEC1	0.64	2	4515	512.3	8.3
IPI00217784	Isoform 4 of Protein NDRG4	NDRG4	6.49	2	339	37	2.71
IPI00374864	Late cornified envelope protein 3B	LCE3B	36.84	4	95	9.8	2.66
IPI00749245	Secreted frizzled-related protein 1	SFRP1	5.1	1	314	35.4	2.52
IPI00908476	Transmembrane BAX inhibitor motif-containing protein 4	TMBIM4	10.12	1	168	19	2.5
IPI00414347	Isoform 2 of Ankyrin repeat and SOCS box protein 18	ASB18	5.26	1	437	47.5	2.15
IPI00885137	Isoform 4 of Sodium channel and clathrin linker 1	SCLT1	18.99	1	79	9.3	2.13
IPI00015842	Reticulocalbin-1	RCN1	8.76	1	331	38.9	0
IPI00909283	Cadherin-11	CDH11	5.13	1	779	85.5	0
IPI00910553	moderately similar to Peroxiredoxin-6	PRDX6	16.83	1	101	11.2	3.9

	Unique proteins induced by *P. gingivalis*LPS_{1690}	Gene	Coverage	Peptides	Amino acids	MW (kDa)	Score
IPI00878107	Interleukin-6	IL-6	6.35	1	252	27.4	3.57
IPI00654838	Isoform 3 of NADPH oxidase activator 1	NOXA1	4.68	1	427	45.2	3.51
IPI00410150	Isoform 1 of Usherin	USH2A	0.6	1	5202	575.2	3.05
IPI00847815	Isoform 3 of Solute carrier family 25 member 35	SLC25A35	8.36	1	287	30.7	2.75
IPI00059240	Insulin gene enhancer protein ISL-2	ISL2	6.13	1	359	39.7	2.45
IPI00001677	Outer dense fiber protein 1	ODF1	9.6	1	250	28.3	2.39
IPI00895907	Rho guanine nucleotide exchange factor 18	ARHGEF18	2.07	1	1015	114	2.28
IPI00456969	Cytoplasmic dynein 1 heavy chain 1	DYNC1H1	0.17	1	4646	532.1	2.2
IPI00024282	Ras-related protein Rab-8B	RAB8B	6.28	1	207	23.6	1.89
IPI00829922	Isoform 2 of Zinc transporter 8	ZNT8	2.81	1	320	35	12.01
IPI00442805	highly similar to Ligatin	DMXL2	1.82	1	440	49.5	11.65
IPI00641216	Putative uncharacterized protein TMCC2	TMCC2	8.59	1	256	28.1	8.97
IPI00216362	Isoform 2 of Otoferlin	OTOF	0.65	1	1230	140.2	3.72
PI00739661	otogelin isoform 2	OTOG	0.32	1	2852	306.8	3.58

Table S3. Differentially regulated secretory proteins in HGFs in response to *P. gingivalis* $LPS_{1435/1449}$ and LPS_{1690} identified by LC-MS/MS approach.

Position	Gene symbol	Gene name	Fold changes	
			$PgLPS_{1435}$	$PgLPS_{1690}$
A01	ALB	Albumin	7.07	14.22
A02	ALOX12	Arachidonate 12-lipoxygenase	2.14	1.07
A03	ANGPTL7	Angiopoietin-like 7	1.2	0.67
A04	AOX1	Aldehyde oxidase 1	1.1	1.27
A05	APOE	Apolipoprotein E	1.29	0.95
A06	ATOX1	ATX1 antioxidant protein 1 homolog (yeast)	1.15	1.05
A07	BNIP3	BCL2/adenovirus E1B 19kDa interacting protein 3	1.15	1.2
A08	CAT	Catalase	1.04	0.93
A09	CCL5	Chemokine (C-C motif) ligand 5	3.19	49
A10	CCS	Copper chaperone for superoxide dismutase	1.18	0.99
A11	CSDE1	Cold shock domain containing E1, RNA-binding	1.02	1.05

A12	CYBA	Cytochrome b-245, alpha polypeptide	1.38	1.22
B01	CYGB	Cytoglobin	1.45	0.99
B02	DGKK	Diacylglycerol kinase, kappa	2.17	0.47
B03	DHCR24	24-dehydrocholesterol reductase	1.58	1.5
B04	DUOX1	Dual oxidase 1	0.51	0.76
B05	DUOX2	Dual oxidase 2	1.75	1.54
B06	DUSP1	Dual specificity phosphatase 1	1.13	1.11
B07	EPHX2	Epoxide hydrolase 2, cytoplasmic	0.81	0.71
B08	EPX	Eosinophil peroxidase	1.88	1.14
B09	FOXM1	Forkhead box M1	1.33	1.17
B10	GLRX2	Glutaredoxin 2	0.91	1.15
B11	GPR156	G protein-coupled receptor 156	0.45	0.67
B12	GPX1	Glutathione peroxidase 1	0.98	0.91
C01	GPX2	Glutathione peroxidase 2 (gastrointestinal)	1.12	0.53
C02	GPX3	Glutathione peroxidase 3 (plasma)	1.07	1.25

C03	GPX4	Glutathione peroxidase 4 (phospholipid hydroperoxidase)	1.13	1.16
C04	GPX5	Glutathione peroxidase 5 (epididymal androgen-related protein)	0.36	0.34
C05	GPX6	Glutathione peroxidase 6 (olfactory)	1.14	1.36
C06	GPX7	Glutathione peroxidase 7	1.13	1.16
C07	GSR	Glutathione reductase	1.44	1.23
C08	GSS	Glutathione synthetase	0.98	0.87
C09	GSTZ1	Glutathione transferase zeta 1	0.95	0.98
C10	GTF2I	General transcription factor II, i	1.47	1.45
C11	KRT1	Keratin 1	1.03	0.72
C12	LPO	Lactoperoxidase	1.61	1.05
D01	MBL2	Mannose-binding lectin (protein C) 2, soluble (opsonic defect)	0.98	0.28
D02	MGST3	Microsomal glutathione S-transferase 3	1.02	1.15
D03	MPO	Myeloperoxidase	2.3	2.03

D04	MPV17	MpV17 mitochondrial inner membrane protein	0.79	0.82
D05	MSRA	Methionine sulfoxide reductase A	0.73	0.88
D06	MT3	Metallothionein 3	3.22	2.5
D07	MTL5	Metallothionein-like 5, testis-specific (tesmin)	0.82	0.77
D08	NCF1	Neutrophil cytosolic factor 1	0.66	0.46
D09	NCF2	Neutrophil cytosolic factor 2	2.04	1.34
D10	NME5	Non-metastatic cells 5, protein expressed in (nucleoside-diphosphate kinase)	1.04	1.29
D11	NOS2	Nitric oxide synthase 2, inducible	1.96	1.1
D12	NOX5	NADPH oxidase, EF-hand calcium binding domain 5	0.45	1.56
E01	NUDT1	Nudix (nucleoside diphosphate linked moiety X)-type motif 1	1.09	0.99
E02	OXR1	Oxidation resistance 1	1.14	1.22
E03	OXSR1	Oxidative-stress responsive 1	1.01	1.05
E04	PDLIM1	PDZ and LIM domain 1	1.68	1.1

E05	IPCEF1	Interaction protein for cytohesin exchange factors 1	0.46	0.52
E06	PNKP	Polynucleotide kinase 3'-phosphatase	1.15	0.99
E07	PRDX1	Peroxiredoxin 1	1	0.99
E08	PRDX2	Peroxiredoxin 2	1.27	1.09
E09	PRDX3	Peroxiredoxin 3	1.03	0.74
E10	PRDX4	Peroxiredoxin 4	1.09	1.12
E11	PRDX5	Peroxiredoxin 5	0.93	0.92
E12	PRDX6	Peroxiredoxin 6	1.11	1.01
F01	PREX1	Phosphatidylinositol-3,4,5-trisphosphate-dependent Rac exchange factor 1	1.13	0.99
F02	PRG3	Proteoglycan 3	2.3	2.03
F03	PRNP	Prion protein	1	1.24
F04	PTGS1	Prostaglandin-endoperoxide synthase 1 (prostaglandin G/H synthase and cyclooxygenase)	1.09	0.92

F05	PTGS2	Prostaglandin-endoperoxide synthase 2 (prostaglandin G/H synthase and cyclooxygenase)	0.68	1.38
F06	PXDN	Peroxidasin homolog (Drosophila)	1.37	1.41
F07	PXDNL	Peroxidasin homolog (Drosophila)-like	2.3	5.83
F08	RNF7	Ring finger protein 7	1.19	1.07
F09	SCARA3	Scavenger receptor class A, member 3	1.27	1.18
F10	SELS	Selenoprotein S	1.25	1.31
F11	SEPP1	Selenoprotein P, plasma, 1	0.89	0.96
F12	SFTPD	Surfactant protein D	1	0.88
G01	SGK2	Serum/glucocorticoid regulated kinase 2	1.09	1.67
G02	SIRT2	Sirtuin (silent mating type information regulation 2 homolog) 2 (S. cerevisiae)	0.91	0.78
G03	SOD1	Superoxide dismutase 1, soluble	1.24	1.43
G04	SOD2	Superoxide dismutase 2, mitochondrial	1.11	16
G05	SOD3	Superoxide dismutase 3, extracellular	1.23	1.64

G06	SRXN1	Sulfiredoxin 1 homolog (S. cerevisiae)	1.05	0.8
G07	STK25	Serine/threonine kinase 25 (STE20 homolog)	0.79	0.7
G08	TPO	Thyroid peroxidase	0.55	0.55
G09	TTN	Titin	1.1	1.11
G10	TXNDC2	Thioredoxin domain containing 2 (spermatozoa)	0.4	0.75
G11	TXNRD1	Thioredoxin reductase 1	1.12	1.26
G12	TXNRD2	Thioredoxin reductase 2	1.06	1.06

Table S4. Differential expression profile of genes associated with oxidative stress and antioxidant defense in HGFs in response to *P. gingivalis* $LPS_{1435/1449}$ and LPS_{1690}.

Primers	Sequence (5'-3')
CTSB-F	AAT GCG CAC GTC AGC GTG GA
CTSB-R	GAC TGG CAC GAC AGG CCC AC
CST3-F	CGC AGC GGG TCC TCT CTA TCT A
CST3-R	CCA TGG TCG GCT AGG ACG CG
PPIA-F	AGG GTG GTG ACT TTA CAC GC
PPIA-R	ATC CAG CCA TTC AGT CTT GG
ADAM 28-F	CAG TTC CTG CAG CCG TCT CAG C
ADAM 28-R	CCC GCA GTG TGG GGC ATG TG
TXN1-F	CTG CTT TTC AGG AAG CCT TG
TXN1-R	TGT TGG CAT GCA TTT GAC TT
TXN2-F	AGC CCG GAC AAT ATA CAC CA
TXN2-R	AAT ATC CAC CTT GGC CAT CA
PRDX1-F	TTT GGT ATC AGA CCC GAA GC
PRDX1-R	TCC CCA TGT TTG TCA GTG AA

PRDX2-F	CCA GAC GCT TGT CTG AGG AT
PRDX2-R	ACG TTG GGC TTA ATC GTG TC
SOD1-F	AGG GCA TCA TCA ATT TCG AG
SOD1-R	TGC CTC TCT TCA TCC TTT GG
MnSOD-F	CTG ATT TGG ACA AGC AGC AA
MnSOD-R	CTG GAC AAA CCT CAG CCC TA
β-actin-F	TTG GCA ATG AGC GGT T
β-actin -R	AGT TGA AGG TAG TTT CGT GGA T

Table S5. Nucleotide sequences of primers used for real-time qPCR
